# Real-time viscoelastic deformability cytometry: High-throughput mechanical phenotyping of liquid and solid biopsies

**DOI:** 10.1126/sciadv.abj1133

**Published:** 2024-12-04

**Authors:** Mohammad Asghari, Sarah Duclos Ivetich, Mahmut Kamil Aslan, Morteza Aramesh, Oleksandr Melkonyan, Yingchao Meng, Rong Xu, Monika Colombo, Tobias Weiss, Stefan Balabanov, Stavros Stavrakis, Andew J. deMello

**Affiliations:** ^1^ Institute for Chemical and Bioengineering, ETH Zürich, 8093 Zürich, Switzerland.; ^2^ Department of Materials Science and Engineering, Uppsala University, Uppsala, Sweden.; ^3^ Department of Information Technology and Electrical Engineering, ETH Zürich, 8092 Zürich, Switzerland.; ^4^ Department of Neurology, University Hospital Zürich, 8091 Zürich, Switzerland.; ^5^ Clinical Neuroscience Center, University of Zürich, 8091 Zürich, Switzerland.; ^6^ Department of Mechanical and Production Engineering, Aarhus University, Aarhus, Denmark.; ^7^ Clinic for Medical Oncology and Hematology, University Hospital Zürich, 8091 Zürich, Switzerland.; ^8^ University Center for Laboratory Medicine and Pathology, University Hospital Zürich, 8091 Zürich, Switzerland.

## Abstract

In principle, the measurement of mechanical property differences between cancer cells and their benign counterparts enables the detection, diagnosis, and classification of diseases. Despite the existence of various mechanophenotyping methods, the ability to perform high-throughput single-cell deformability measurements on liquid and/or solid tissue biopsies remains an unmet challenge within clinical settings. To address this issue, we present an ultrahigh-throughput viscoelastic microfluidic platform able to measure the mechanical properties of single cells at rates of up to 100,000 cells per second (and up to 10,000 cells per second in real time). To showcase the utility of the presented platform in clinical scenarios, we perform single-cell phenotyping of both liquid and solid tumor biopsies, cytoskeletal drug analysis, and identification of malignant lymphocytes in peripheral blood samples. Our viscoelastic microfluidic methodology offers opportunities for high-throughput, label-free single-cell analysis, with diverse applications in clinical diagnostics and personalized medicine.

## Teaser

A microfluidic platform measures single-cell mechanical properties of both liquid and solid tumor biopsies at 10,000 cells/s.

## INTRODUCTION

In recent years, the use of traditional (excisional or incisional) biopsies in the diagnosis or monitoring of cancers has been supplemented or even replaced by liquid biopsies, where bodily fluids are sampled and assayed for cancer cells or nucleic acids released by cancers cells. Liquid biopsies are minimally invasive and, in principle, allow the extraction of tumor-derived information in a direct and rapid manner. A key part of the liquid biopsy workflow is the molecular characterization of sampled cells, with the intention of identifying novel biomarkers able to detect or predict disease state (*1*). Although numerous methods (primarily based on cell surface expression) have been developed to isolate cells, their use in cancer diagnostics has been limited by the simple fact that cancer cells are heterogeneous with respect to marker expression (*2*). Alternatively, it is well known that cells are viscoelastic entities, with their physical and mechanical properties, such as size, shape, and deformability, varying in response to stresses associated with diverse physiological and disease processes (*3*). Expectedly, cell deformability has been proposed as a label-free biomarker to enhance both disease diagnosis and clinical decision-making (*4*, *5*). This is especially relevant for cancer, because cancerous cells are typically more deformable than noncancerous cells and can therefore be differentiated from their healthy counterparts through measurement of mechanical properties (*6*–*10*). While the origins of cell deformability variations are not fully understood, the ability to measure such properties at the single-cell level would undoubtedly enhance disease diagnostics/prognostics. In this regard, several analytical tools have shown utility in probing the mechanical properties of single cells. These include atomic force microscopy (*11*), gradual micropore filtering (*12*), micropipette aspiration (*13*), optical stretching (*14*), and magnetic tweezers (*15*). While these methods do allow measurement of cell deformability, typical workflows are complex and analytical throughputs (of a few cells per second) are unacceptably low, which, in turn, has limited their use in clinical settings. Fortunately, microfluidic techniques have begun to address these limitations, offering the promise of mechanical phenotyping in a robust, rapid, and label-free manner (*16*). Microfluidic systems for deformability cytometry leverage either physical structures or fluid flow to induce cellular deformation, with constriction-based deformability cytometry (cDC) (*17*–*20*), shear flow deformability cytometry (sDC) (*21*–*25*), and extensional flow deformability cytometry (xDC) (*5*, *26*, *27*) being the most important methods reported to date. Constriction-based deformability cytometry involves forcing cells through a constriction that is smaller than their diameter, with deformability being inferred from the transit time through the constriction. While simple to implement, cells are in continuous contact with channel walls [and are thus prone to blocking (*20*)] and throughput is limited to approximately one cell/s. In contrast, both shear flow deformability cytometry and extensional flow deformability cytometry operate in a contactless manner, using microfluidic channels with cross-sectional dimensions greater than the diameter of the cells under study. While hydrodynamic forces are used to deform cells in both cases, with deformability being calculated via image analysis, xDC and sDC leverage quite different microfluidic geometries. In xDC, cells are stretched by placing them in a cross-slot geometry at the point where two counter propagating fluids meet (*26*). Because cells are delivered to the junction at several meters per second and fully decelerated (and deformed) within a few microseconds, mechanical phenotyping at throughputs in excess of 1000 cell/s can be realized. Conversely, in sDC, cells moving along a narrow microfluidic channel are focused into a single file using a sheath flow (*21*). Shear forces and pressure gradients deform cells into characteristic “bullet” shapes without contacting the channel walls. sDC can operate at throughputs up to 1000 cell/s but, as with conventional flow cytometry, consumes excessively large sample and reagent volumes due to its reliance on sheath flows.

Recently, viscoelastic fluids have been used to good effect in a range of cell manipulations (*28*–*33*). Viscoelastic fluids are prepared by simply dissolving biological or synthetic polymers in a Newtonian fluid. For example, polyethylene oxide (PEO) has been extensively used to form viscoelastic fluids for cell manipulations in microfluidic devices (*30*). PEO has a molecular weight between 100 and 4000 kDa, with its solutions being strongly viscoelastic and having weak to mild shear-thinning effects (*34*). An important advantage of using viscoelastic fluids in microfluidic systems is their ability to align micrometer-sized species into a single file at the center of a microfluidic channel, without the need for a sheath flow (*33*, *35*). In addition, elastic forces and shear stresses can also be used to deform cells within microfluidic environments (*36*, *37*). As noted, sDC and xDc use single-channel configurations with channel widths being slightly larger than the cells being processed, and can be operated at rates of up to 1000 cell/s. Analytical throughput is most easily enhanced through device parallelization. Unfortunately, parallelizing sDC platforms is challenging due to the presence of multiple sheath flows, while parallelization of an xDC platform is hindered by the use of channel cross-slot geometries. Although sDC is capable of performing real-time deformability analysis, a maximum throughput of 100 cell/s is two orders of magnitude lower than that achievable using conventional flow cytometry (*38*). Recently xDC has emerged as a promising technology with substantial potential in clinical diagnostics. Specifically, its integration into Food and Drug Administration–cleared diagnostic tests, such as the IntelliSep platform, was recently demonstrated by Cytovale (*39*, *40*). These developments underscore the potential impact of xDC in enhancing diagnostic utility through precise single-cell deformability analysis. That said, a diagnostic device for whole blood analysis based on cell deformability measurements, which improves upon existing methods in terms of throughput and operational simplicity, is still lacking.

Herein, we present a (real-time) deformability cytometer that addresses this gap by enhancing throughput via parallelization and minimizing device blocking, making it suitable for biopsy-based diagnostics. Our cytometer uses viscoelastic fluids to focus and deform cells without the need for sheath fluids and operates at throughputs between 10,000 and 100,000 cell/s through channel parallelization. We term the technique viscoelastic deformability cytometry or vDC because viscoelastic forces are used to both focus and deform cells. High-resolution bright-field images of large numbers of single cells moving through the deformation zone are recorded using a simple optical microscope and a CMOS image sensor. Real-time image analysis and processing is used to quantify deformability, providing for mechanical phenotyping in a rapid and label-free manner. We first validate the vDC platform by measuring differences in the mechanical properties of blood cells and two breast cancer cell lines (BT474 and MDA-MB468). Platform sensitivity is assessed by detecting changes in Jurkat cell deformability that result from cytoskeletal-perturbing drug treatments. To showcase the ultrahigh-throughput capability of the deformability cytometer, we performed a rare cell detection experiment in whole blood that mimics the analysis of circulating tumor cells (CTCs) in blood. Next, the potential clinical utility of vDC is assessed through the analysis and characterization of solid tumor-derived cancer cells. Specifically, we investigate mechanical differences between two types of patient-derived glioma cells, namely, glioma-initiating cells (GICs) that have stem cell–like properties and are resistant to chemotherapy and radiotherapy and more differentiated long-term serum-cultured (LTC) cells. Last, we showcase the utility of vDC in liquid biopsy applications through the rapid and sensitive detection of chronic lymphocytic leukemia (CLL). Our vDC platform is simple in construction, high throughput in nature, and able to perform label-free analysis of heterogeneous cell populations in a range of research and clinical settings.

## RESULTS

**Fig. 1.**
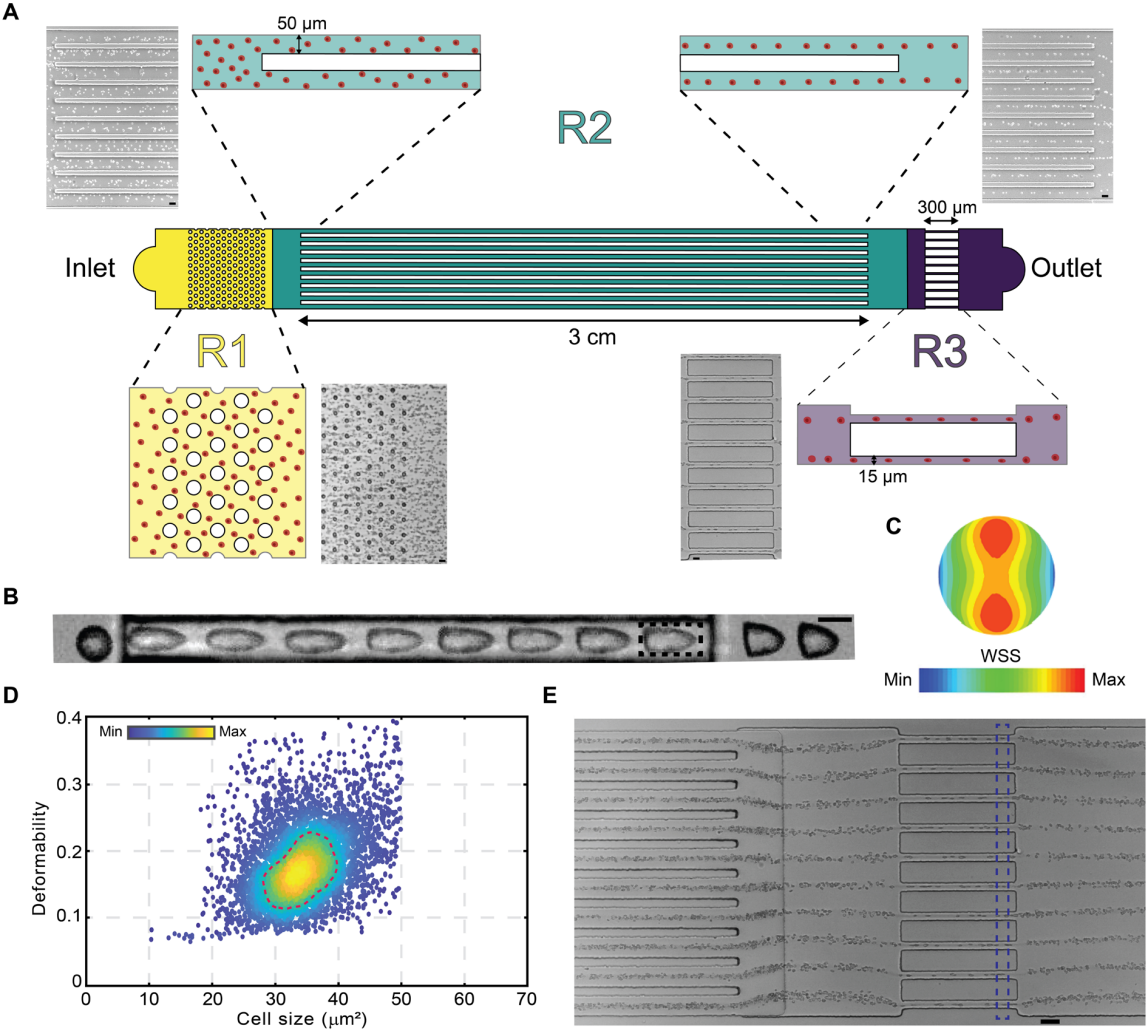
Image-based viscoelastic deformability cytometry (vDC). (**A**) Randomly distributed cells are introduced into the focusing region via the inlet. As cells move downstream, they are focused around the channel centerline due to elastic forces. Focused cells then enter a constricted channel, where they are deformed. After the cells have transited the constriction, they relax before exiting at the outlet (scale bar, 25 μm). (**B**) Bright-field images of a cell moving through the deformation region showing the progression of deformation as a function of distance (scale bar, 10 μm). In subsequent experiments, images of deformed cells are acquired in each channel in the region marked by the dotted box. (**C**) Shear stress distribution around a representative cell inside the constriction channel. Surface color indicates the magnitude of the shear stress. (**D**) A representative scatterplot reporting deformability against cell size for a population of 10,000 red blood cells. Color defined according to a linear density scale; dotted line defines the 50% density contour. (**E**) Focusing and deformation of blood cells in a vDC microfluidic device consisting of an array of 10 parallel channels (scale bar, 30 μm).

Figure 1A presents a schematic of the viscoelastic deformability cytometer. The microfluidic device is simple in construction, having three primary functions. The first is to remove cell aggregates from the sample using a pillar array and prevent clogging of the device (R1). The second is to align and focus cells moving within an array of parallel microfluidic channels into a narrow stream around the centerline (R2), and the third is to deform these cells as they transit the deformation zone (R3) (movie S1). In the focusing zone, cells flowing along a microfluidic channel are focused laterally and vertically due to the interplay between elastic (originating from the viscoelastic carrier fluid) and inertial forces (text S1 and fig. S1). The focusing of cells in the vDC device occurs in both horizontal and vertical directions, resulting in three-dimensional focusing. Quantification of focusing performance has been described previously, where confocal microscopy was used to measure the position and distribution of cells at various positions along the *z* axis of a microfluidic channel (*35*). To confirm that cell focusing occurs within the 3-cm-long square cross-sectional channel, we acquired images of Jurkat cells (having an average size of 11 μm) and 8-μm beads, at several distances downstream of the inlet (fig. S2). As can be seen, cell focusing already occurs at a distance of 2 cm from the inlet. The selection of a 3-cm channel length is based on the observation that smaller cells, typically found in a blood sample, tend to focus at the channel center more slowly than larger cells, necessitating a longer channel length. As the focused cells enter and flow through the array of the constricted channels, they experience high stresses that cause them to deform. Each microchannel within the focusing zone is 3 cm long and has a square cross section (50 μm by 50 μm). Three centimeters is sufficiently long so as to ensure efficient cell focusing (*28*). Each channel in the deformation zone is 15 μm wide, 15 μm deep, and 300 μm long. Within such constricted channels, suspended cells are subject to both shear and normal stresses because the flow velocity is higher toward the center of the channel when compared to regions closer to the channel walls. Such stresses cause cells to deform and adopt “bullet-like” shapes, as seen in bright-field images taken in the deformation region (Fig. 1B) and as predicted by computational fluid dynamics (CFD) simulations (Fig. 1C). We also performed CFD simulations to analyze the impact of fluid properties and velocity on wall shear stress (WSS). Figure S3A reports flow streamlines around a 6-μm-diameter cell with a mean fluid velocity of 0.35 m/s, while fig. S3B presents the mean WSS on the cell surface and a cross section of the WSS distribution on the cell (dotted box) within a viscoelastic medium containing 1% PEO, at the same mean fluid velocity. For 0.1% PEO, increasing the pressure from 500 to 2000 mbar raises the mean WSS on the cell from 1116.5 to 8799.14 Pa (fig. S3, C and D). A similar trend is observed for higher PEO concentrations. For example, when using PEO concentrations ranging from 0.1% to 1.0% w/v, the WSS experienced by the cell increases from 1116.5 to 15,347.5 Pa for pressures between 500 and 2000 mbar (fig. S3D). Accordingly, both experimental conditions, such as inlet pressure, and the rheological properties of the fluid, such as polymer concentration, substantially affect the WSS on the cell, leading to deformation.

To capture blur-free images, and assuming an average cell velocity of 0.7 m/s, exposure times were set to 2 μs with a recording rate of either 1000 or 10,000 frames/s. Images of single cells at the end of the deformation region (identified by the dashed box in Fig. 1B) are then used to measure the cell area, *A*, the cell perimeter, *l*, and the deformability, *D*, according to

$$D=1-2\sqrt{\pi A}/l$$ (1)

Subsequently, scatterplots reporting cell deformability versus size can be constructed, as shown in Fig. 1D. As previously noted, because vDC does not require the use of a sheath flow to focus cells, multiplexing can be realized by simply arranging multiple deformation microchannels in parallel and simultaneously observing cells in equivalent regions of each channel (Fig. 1E). Here, to ensure efficient imaging of every single cell within a sample, the horizontal size of the region of interest (ROI) was set to be slightly larger than the maximum dimension of a deformed cell (Fig. 1E, dashed blue rectangle). In this manner, cells can be imaged and analyzed at exceptionally high frame rates. In real-time mode, a maximum throughput of 10,000 cells/s was achieved when using a 20× objective at a rate of 1000 frames/s. In off-line mode at the same magnification and at a rate of 10,000 frames/s, the vDC platform provides for a maximum throughput of approximately 100,000 cells/s. Both throughput values are two orders of magnitude higher than accessible using the current state-of-the-art methods (*41*).

To assess the impact of the PEO solution on cell viability during vDC analysis, we performed viability measurements on cells before and after the deformation process. Specifically, HEK (human embryonic kidney cells), K562 (human chronic myelogenous leukemia cell line), and peripheral blood mononuclear cells (PBMCs) were tested. Figure S4 illustrates the viability of HEK, K562, and PBMCs collected after passage through the deformability device. The viability of cells after vDC processing is not statistically different to cells that had not been processed, indicating that both the PEO solution and passage through the channel constriction do not significantly affect cell viability.

Figure S5 reports the dependence of cellular velocity within the deformation channels on PEO concentration, PEO molecular mass, and inlet pressure. Here, the velocity of single Jurkat cells (at the end of the deformation zone) was determined by analysis of bright-field images. Velocities were evaluated for PEO concentrations of 0.1, 0.5, and 0.8%; pressures of 500, 1000, 1500, and 2000 mbar; and PEO molecular masses of 1, 2, and 5 MDa. As can be seen, the average flow velocity varied widely, between 5 and 200 cm/s. More specifically, the flow velocity decreased with increasing PEO concentration and molecular mass, while it increased as a function of inlet pressure.

In addition, we investigated the effect of inlet pressure on the focusing of both beads and cells. Specifically, we examined the focusing behavior of 8-μm-diameter beads (at a concentration of 10^6^/ml) and Jurkat cells (at a concentration of 10^5^/ml) across a range of flow rates as characterized by the Reynolds (Re) and Weissenberg (Wi) numbers. Figure S6 presents an image stack of 20 snapshots taken at the end of the focusing region for various Re and Wi values. Excellent focusing of both Jurkat cells (fig. S6A) and 8-μm beads (fig. S6B) is achieved using inlet pressures up to 1500 mbar. Accordingly, the applied pressure can be varied over a wide range without any detrimental effects on the focusing efficiency. However, increasing the inlet pressure to 2000 mbar resulted in the defocusing of both particles and cells.

In addition, we examined the dependence of cell deformability (5000 Jurkat cells examined) on the inlet pressure. Figure S7 indicates that higher inlet pressures lead to increased cell deformability. Specifically, when using 1 MDa and 0.1% PEO, mean deformability values of 0.030, 0.050, 0.065, and 0.085 were extracted for inlet pressures of 500, 1000, 1500, and 2000 mbar, respectively. We also simulated the cell deformation process in a viscoelastic medium using the fluid-solid interaction (FSI) model, which is implemented in COMSOL Multiphysics 6.0 (text S2). The viscoelastic properties of different PEO solutions used as an input for the simulations were extracted from rheological experiments that measure the viscosities of PEO at different shear rates (fig. S8, table S1, and text S3). These simulations were used to extract the relationships between the deformability and velocity, viscosity, and shear stress in PEO viscoelastic fluids.

Figure S9 illustrates the impact of cell velocity on its deformation and more specifically how the cell shape changes at each flow rate and the corresponding deformability values derived from the simulation. As the cell velocity increases from 0.1 to 0.75 m/s, deformability changes significantly from 0.003 to 0.111 and the cell shape transitions from circular to bullet-like (text S4). In addition, we analyzed, in our simulations, the effect of fluid viscosity on cell deformation by implementing the viscosities of 1 MDa PEO concentrations of 0.1, 0.5, 0.8, and 1.0% w/v (text S5 and fig. S10). The simulations showed that increasing fluid viscosity can result in a two-order magnitude increase in deformability. In addition, through simulations, we investigated the effect of cell size on cell deformability (text S6 and fig. S11). These simulations predicted that as the cell size increases from 5 to 14 μm, deformability increases significantly from 0.0025 to 0.053.

To assess whether PEO causes osmotic swelling in cells, we conducted flow cytometry experiments, comparing the forward scatter (FSC) profiles of cells in phosphate-buffered saline (PBS) buffer in the absence and presence of PEO of varying molecular weights (1, 2, and 5 MDa) and concentrations (0.1 to 0.8%) (fig. S12A). Cell size, as indicated by the FSC signal, appears to be inversely proportional to the molecular weight and concentration of PEO (fig. S12B). This suggests that higher viscosity reduces oxygen availability, causing cells to shrink to conserve resources. We also observed an increase in the (SD) values of FSC signals with higher PEO concentrations, indicating that such increased variability likely results from difficulties in obtaining precise measurements in thicker fluids. As shown in fig. S12C, the median FSC intensity difference between the 0.1% w/v 1 MDa PEO sample (used in all experiments) and the control is not statistically significant, demonstrating that a 0.1% w/v concentration of 1 MDa PEO has a negligible effect on osmotic swelling. In addition to influencing cell osmolality, PEO can also affect the osmolality of the buffer, which, in turn, can affect the mechanical properties of deformable cells (text S7 and fig. S13). Although we found that the use of 1 MDa PEO at a 0.1% w/v concentration affects the buffer osmolality (fig. S13B), this does not affect cell size, as demonstrated by the FSC profiles of cells (fig. S12).

### Blood and breast cancer cell line phenotyping

**Fig. 2.**
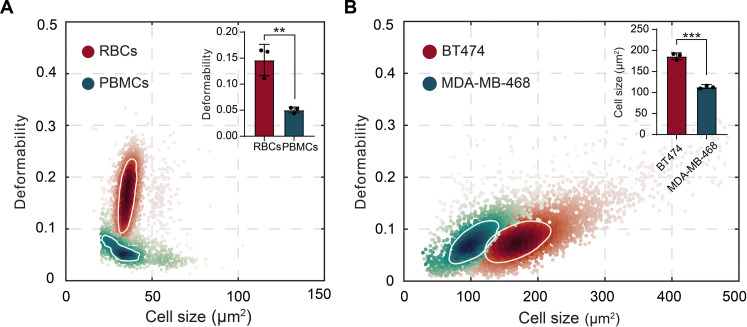
Mechanical phenotyping of blood cells and breast cancer cells. (**A**) Density scatterplot of RBCs and PBMCs (measured in separate experiments) using a pressure of 1 bar within a 15 μm by 15 μm cross-sectional channel. The white loops correspond to 50% density contour lines. Although both subpopulations present similar size properties, they can be discriminated through measurements of deformability (*P* < 0.0021) when performing a statistical comparison using a nonparametric *t* test. (**B**) Density scatterplot of two breast cancer cell lines (BT474 and MDA-MB-468) measured separately using an inlet pressure of 1 bar within a 15 μm by 15 μm cross-sectional channel. The white loops correspond to 50% density contour lines. Cell lines display similar deformability values but can be discriminated through measurement of size (*P* < 0.0002) after performing a statistical comparison using a nonparametric *t* test. The height of each bar represents the average of three measurements. Approximately 5000 cells were analyzed on each experiment.

To begin to assess the clinical capabilities of vDC, we initially examined red blood cells (RBCs) and PBMCs contained in a 20× diluted blood sample (see Materials and Methods for details regarding the isolation process). Samples were injected into the microfluidic device at a pressure of 1 bar. Data for each cell type were acquired individually and are presented in Fig. 2A, with a minimum of 5000 cells being probed in each experiment. Although the two cellular subpopulations have similar average sizes, 35.4 ± 2.3 μm^2^ for RBCs and 28.1 ± 4.9 μm^2^ for PBMCs, they can be readily distinguished based on differences in deformability (*P* < 0.0021). Specifically, RBCs exhibited an average deformability of 0.16 ± 0.03 with PBMCs exhibiting an average deformability of 0.05 ± 0.01.

Next, we used vDC to mechanically phenotype two breast cancer cell lines: BT474 and MDA-MB468. Both BT474 and MDA-MB468 cells exhibit an epithelial morphology, with BT474 cells being isolated from a solid, invasive ductal breast carcinoma and MDA-MB468 cells being extracted a pleural effusion of mammary gland and breast tissue. Again, cells were injected at a pressure of 1 bar and their passage through a deformation microchannel was imaged to extract both size and deformability information (Fig. 2B). Unlike the experiments with blood cells, the cell lines exhibit almost identical average deformability values, 0.06 ± 0.02 for MDA-MB468 cells and 0.08 ± 0.02 for BT474 cells, with discrimination now being achieved through the assessment of cell area, i.e., 103 ± 16.5 μm^2^ for MDA-MB468 cells and 170 ± 22.4 μm^2^ for BT474 cells (*P* < 0.0002).

### Effects of pharmacological agents on cell deformability

**Fig. 3.**
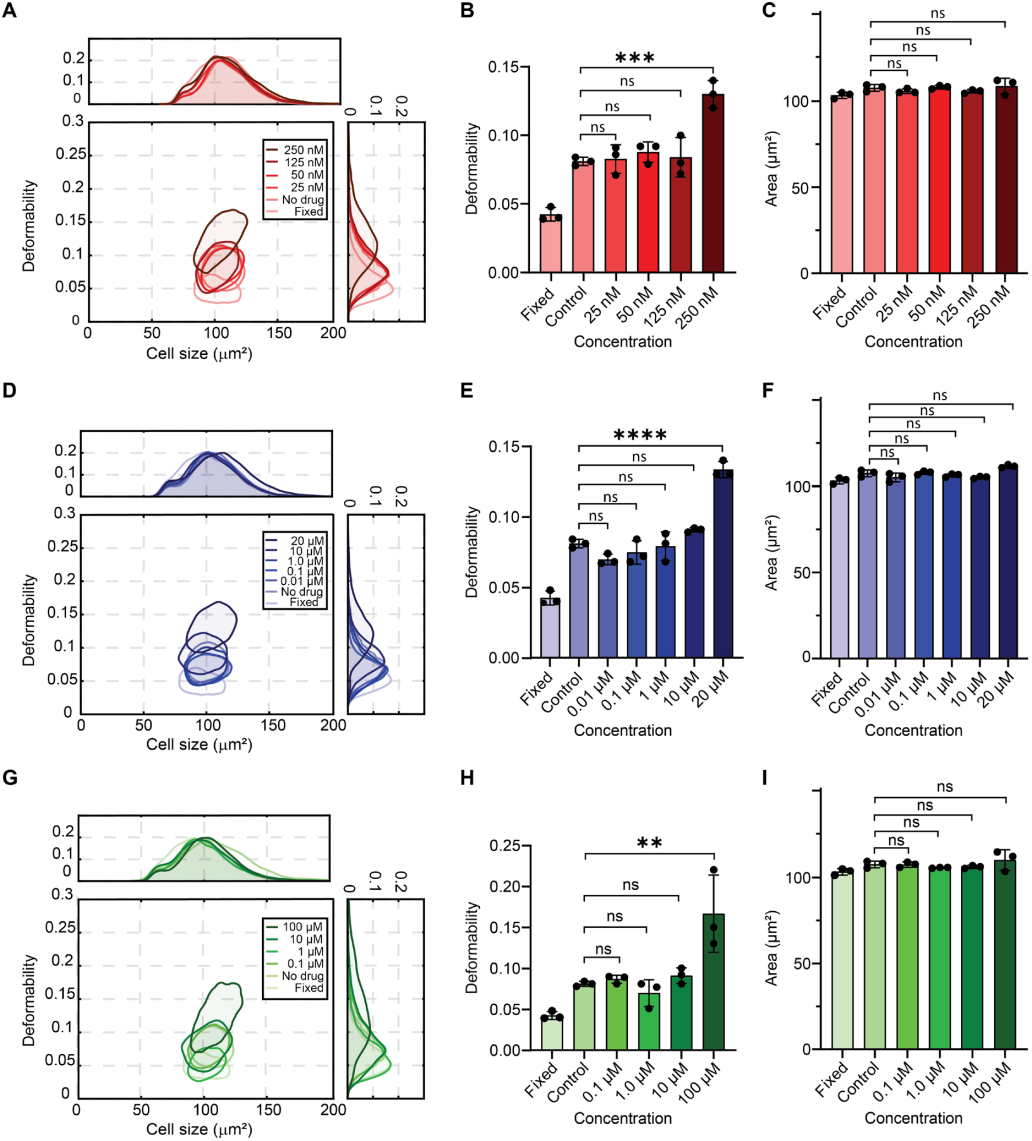
Effect of cytoskeletal drugs Lat B, Cyto D, and Noco on cell deformation. Contour plots (50% density) reporting deformability versus cell size for fixed Jurkat cells and cells incubated without (control) and with different concentrations of cytoskeletal drugs (**A**, **D**, and **G**). Measurements of deformability (**B**, **E**, and **H**) and cell area (**C**, **F**, and **I**) for Jurkat cells exposed to different drug concentrations. Each displayed point represents the mean of 5000 single-cell measurements, with error bars representing 1 SD. Lat B, Cyto D, and Noco concentrations of 250 nM, 20 μM, and 100 μM, respectively, induce significant deformability variation. Statistical comparisons between samples were performed using a one-way ANOVA. The height of each bar represents the average of three measurements. In all cases, the mean cell size difference between control and drug-treated cells is not statistically significant. Conversely, for 250 nM Lat B (*P* = 0.0003), 20 μM Cyto D (*P* < 0.0001), and 100 μM Noco (*P* = 0.0033), deformability is significantly different from the control sample.

We next used vDC to investigate the effects of various pharmacological agents on the physical and mechanical properties of cells. Previous studies have demonstrated how actin and microtubule networks control the mechanical properties of cells (*42*, *43*). Actin is a structural protein that forms microfilaments in the cell cytoskeleton (*41*). Microtubules are long, rigid cylindrical polymers of tubulin that resist contractile force and interact with other cytoskeletal polymers to stabilize the cytoskeleton (*44*). Accordingly, we assessed the impact of three cytoskeletal (perturbing) drugs on cell deformability: Latrunculin B (Lat B) and Cytochalasin D (Cyto D), are both potent inhibitors of actin polymerization, and Nocodazole (Noco), is an antimitotic agent that reversibly interferes with microtubule polymerization (*45*, *46*). For all experiments, an inlet pressure of 500 mbar was used, with at least 5000 cells being analyzed per experiment. Contour lines at 50% of the maximum density facilitated comparison between experiments. Lat B concentrations were varied between 25 nM and 250 nM and compared with “fixed cell” and “no-drug” control measurements. Figure 3A displays deformability/cell size contour plots for various Lat B drug concentrations, “no drug,” and fixed cells. Comparison of the contour plots highlights a significant difference in deformability between fixed and viable cells. Figure 3 (B and C) reports the mean values of cell deformability and cell area. Measurements were performed in triplicate for each condition, with the vDC platform being loaded with samples containing cells at four different drug concentrations. Statistical analysis of the replicates reveals that samples exposed to Lat B concentrations up to 125 nM did not show a significant shift in cell deformability when compared to the control. However, exposure to Lat B at a concentration of 250 nM resulted in a significant shift to elevated deformability values, as confirmed by analysis of variance (ANOVA), which yielded a *P* value of 0.0003. When considering cell size (area), no significant change was observed between drug treatment conditions (*P* = 0.88 to 0.99). A similar study of the effects of Cyto D concentration on actin depolymerization was also performed, with Fig. 3D displaying deformability/cell size contour plots for various Cyto D concentrations, no drug (control), and fixed cells. Similarly, data indicate no significant shift in cell deformability for samples exposed to Cyto D concentrations up to 10 μM when compared to the control (Fig. 3E). However, for concentrations at 20 μM, deformability is significantly enhanced (*P* < 0.0001), indicating efficient actin depolymerization. As with Lat B, no significant change in cell size was observed between all treatment conditions (*P* > 0.033). Last, viable cells and cells exposed to Noco showed higher deformability values when compared to fixed cells (Fig. 3G). The exposure of cells to Noco at concentrations up to 10 μM did not yield a significant change in average deformability (Fig. 3H; *P* = 0.9903). However, exposure to 100 μM Noco yielded enhanced deformability values (*P* = 0.0033), with no appreciable change in cell size (Fig. 3I; *P* = 0.87 to 0.99). These data confirm reduced microtubule polymerization upon exposure to Noco.

### Quantification of rare cells in blood samples

As previously noted, high-throughput operation can be particularly important when detecting rare cells in complex samples, such as blood. For example, the accurate quantification of CTCs has considerable diagnostic utility but is challenging due to their low abundance. In this regard, flow cytometry is not ideal, with the analysis of 1 ml of blood (containing many billions of cells) being a time-consuming process, often taking more than 24 hours to complete. This limitation makes flow cytometry impractical for identifying CTCs in diagnostic scenarios. To address this challenge and to showcase the high-throughput nature of our viscoelastic deformability cytometer, we performed single-cell deformability measurements to identify CTCs mimics in blood. Specifically, we used patient-derived human brain glioblastoma cells (LN229) as model CTCs. The LN229 cancer cell line, obtained from the University Hospital Zurich, was chosen for its recognized representativeness of CTCs found in patients with brain glioblastoma (*47*).

**Fig. 4.**
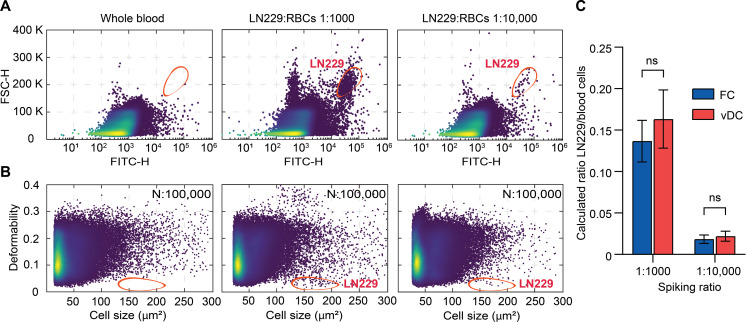
Rare cell quantification using conventional FC and vDC. LN229 cells were spiked in 10-fold diluted blood at ratios of 1:1000 and 1:10,000. (**A**) A FSC-A versus FITC plot of non–cell-spiked blood shows the distribution of RBCs as the major component (left). FSC-H versus FITC-H plots show that LN229 can be distinguished at spiking ratios of LN229/RBCs: 1:1000 (middle) and 1:100,000 (right). (**B**) Scatterplots of deformability versus cell size of the same blood samples as in (A) of 100,000 cells show the ability of vDC to quantify the number of LN229 cells. (**C**) Analyzed ratios measured by conventional FC (blue) and real-time vDC (red) show a close correspondence. The height of each bar represents the average of three measurements. Statistical comparisons between samples were performed using a nonparametric *t* test. The mean ratios of LN229/RBCs between FC and vDC measurements are not significant (ns).

As a gating strategy for detecting “rare” LN229 cells in blood, we first ran a sample of LN229 cells in PBS buffer at a concentration that mimics in vivo concentrations (3.5 cells/μl as measured by flow cytometry, table S2) through the vDC device. We then gated the LN229 cell population from the scatterplot of deformability versus cell size (fig. S14). Subsequently, whole blood was diluted to 10^7^ cells/ml and LN229 cells were spiked in at ratios of 1:1000 and 1:10,000 (LN229: RBCs). We then performed FC and vDC experiments to quantify the number of LN229 cells at these spiking ratios. FSC-H signals at 488 nm were used to assess cell size. Autofluorescence emission at 530 nm (FITC-H) from LN229 cells allowed their discrimination from other blood cells. The exact number of LN229 cells was extracted from two-dimensional density plots of FSC-H versus FITC-H for a nonspiked blood sample (control, Fig. 4A, left) and two samples containing 1:1000 (Fig. 4A, middle) and 1:10,000 (Fig. 4A, right) LN229:RBCs ratios.

LN229-spiked blood samples were injected into the microfluidic platform and subjected to cell deformation. Under standard operating conditions, it takes approximately 1230 s to process a 1.1-ml sample of 10-fold diluted blood, which translates to a throughput of approximately 100,000 cells/s. By analyzing a total of 100,000 cells, LN229 cells were identified (Fig. 4B, middle and right) using the aforementioned gating strategy and a nonspiked blood sample as the control (Fig. 4B, left). Given the low abundance of LN229 cells, we also analyzed 200,000 cells to identify a significant cluster of LN229 cells (fig. S15). Data from flow cytometry (blue bar) and real-time vDC (red bar) showed close correspondence in terms of cell concentrations (Fig. 4C and table S2). However, it is critical to note that in this comparative analysis, conventional flow cytometry experiments were performed using a flow rate of 30 μl/min (required for robust operation) and a 1000× dilution factor (to prevent cartridge blockage and coincident event detection). Notably, vDC was able to quantify LN229 cells as rare as 100 CTCs per million RBCs, using a volumetric flow rate of 80 μl/min and a 10× dilution factor. To showcase the advantages of using the high-throughput device, we performed cell deformability measurements using 20-fold diluted blood and constriction microchannel cross sections of 15 μm by 15 μm. As shown in fig. S16, the scatterplot of approximately 400,000 cells consists of RBCs and WBC subpopulations.

### Phenotyping of patient-derived glioma cells with cancer stem-cell properties versus more differentiated glioma cells

**Fig. 5.**
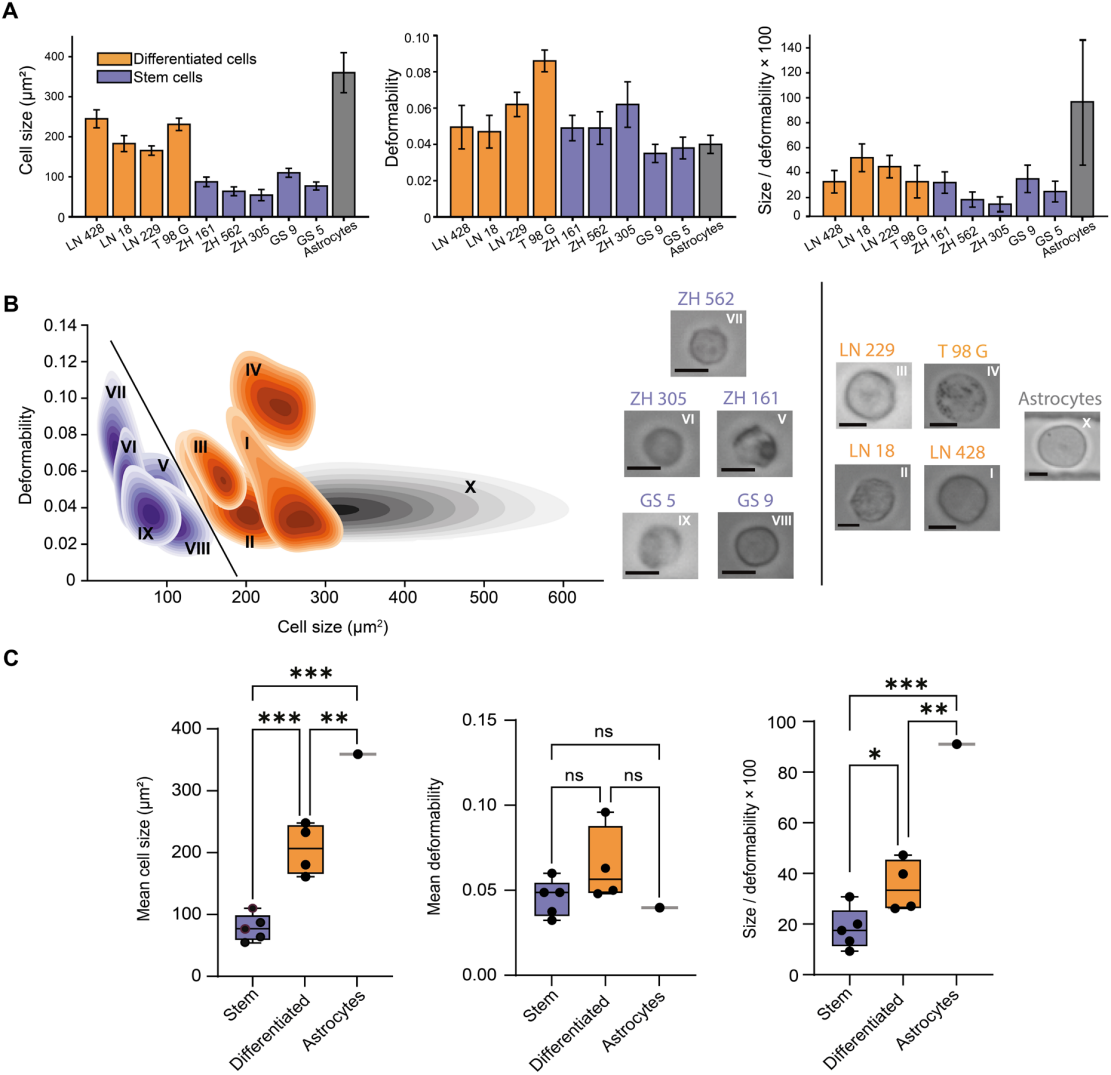
Investigation of glioblastoma stem and differentiated cell signatures using viscoelastic deformability cytometry. (**A**) Bar charts reporting mean cell size, deformability, and size-to-deformability ratio of differentiated and stem-type glioma cancer cells. Error bars represent 1 SD derived from three independent experiments. (**B**) All nine types of glioma cells can be identified on the basis of joint Kernel density estimate plots of deformability versus cell size. The solid line separates the two types of glioma cell lines, i.e., GIC cells (purple) and LTC cells (orange). Control cells (astrocytes: dark gray) exhibit greater heterogeneity with respect to cell size compared to the solid tumor-derived glioma cells. Bright-field images of both differentiated- and stem-type glioma cells allow extraction of features (such as size and deformability) for multidimensional analysis (scale bar, 10 μm). (**C**) Box plots of size, deformability, and size-to-deformability ratio for glioma cell subtypes (GIC: purple, LTC: orange) and a control cell line (dark gray). The horizontal line represents the mean value. Box plot whiskers range from the 5th to 95th percentiles and the length of the whiskers is restricted to a maximum of 1.5 times the interquartile range. Statistical comparisons between these samples were performed using a one-way ANOVA. Mean cell size difference of stem versus differentiated (*P* = 0.0009), stem versus astrocytes (*P* = 0.0001), and differentiated versus astrocytes (*P* = 0.0059). The mean deformability value among the above samples is not significant (ns). Mean cell size-to-deformability ratios of stem versus differentiated cells (*P* = 0.0476), stem cells versus astrocytes (*P* = 0.0003), and differentiated cells versus astrocytes (*P* = 0.0018) show that these three different categories are statistically different.

Glioblastoma is the most common and most aggressive primary brain tumor in adults (*48*). Different glioblastoma cancer cell subpopulations have been identified, ranging from GICs to differentiated glioma cells (*49*, *50*). Patient-derived GIC lines that have stem cell–like properties (including tumor-initiating potential) (*51*), are cultured in serum-free conditions, and are more resistant to chemotherapy and radiotherapy compared to more long-term differentiated glioma cell lines (LTCs) that are cultured under serum-containing conditions (*50*, *52*, *53*). The ability to accurately identify GICs remains an important challenge because of their plasticity and a lack of selective markers (*54*, *55*). In this regard, it is important to note that while some surface markers or combinations thereof have been associated with cancer stem cell–like properties, they are not specific to cancer stem cells (*56*). Accordingly, we used vDC to characterize the mechanical properties of GICs and LTCs and compare them to nonmalignant astrocytes. As shown in Fig. 5A, the mean sizes of GIC cells (specifically ZH-161, ZH-562, ZH-305, GS9, and GS5 stem cells) are significantly smaller than the mean sizes of LTC cells (LN-428, LN-18, LN-229, and T98G). In addition, all glioma cells are smaller than primary human astrocytes (control). While deformability across cell lines is relatively heterogeneous (Fig. 5A, middle), both size and size-to-deformability ratio can be used to differentiate between GIC (stem-like) and LTC (differentiated) cells (Fig. 5A, left and right). In addition, kernel density estimation plots of mean cell deformability against cell size reveal three clusters reporting GIC, LTC, and primary astrocyte populations (Fig. 5B, left). Representative bright-field images of each cell subtype are shown in Fig. 5B (right). Last, it should also be noted that cells originating from both tumor types had significantly lower mean cell size and size-to-deformability ratio values when compared to their healthy counterparts (Fig. 5C).

### Mechanical phenotyping of chronic lymphocytic leukemia cells using vDC

**Fig. 6.**
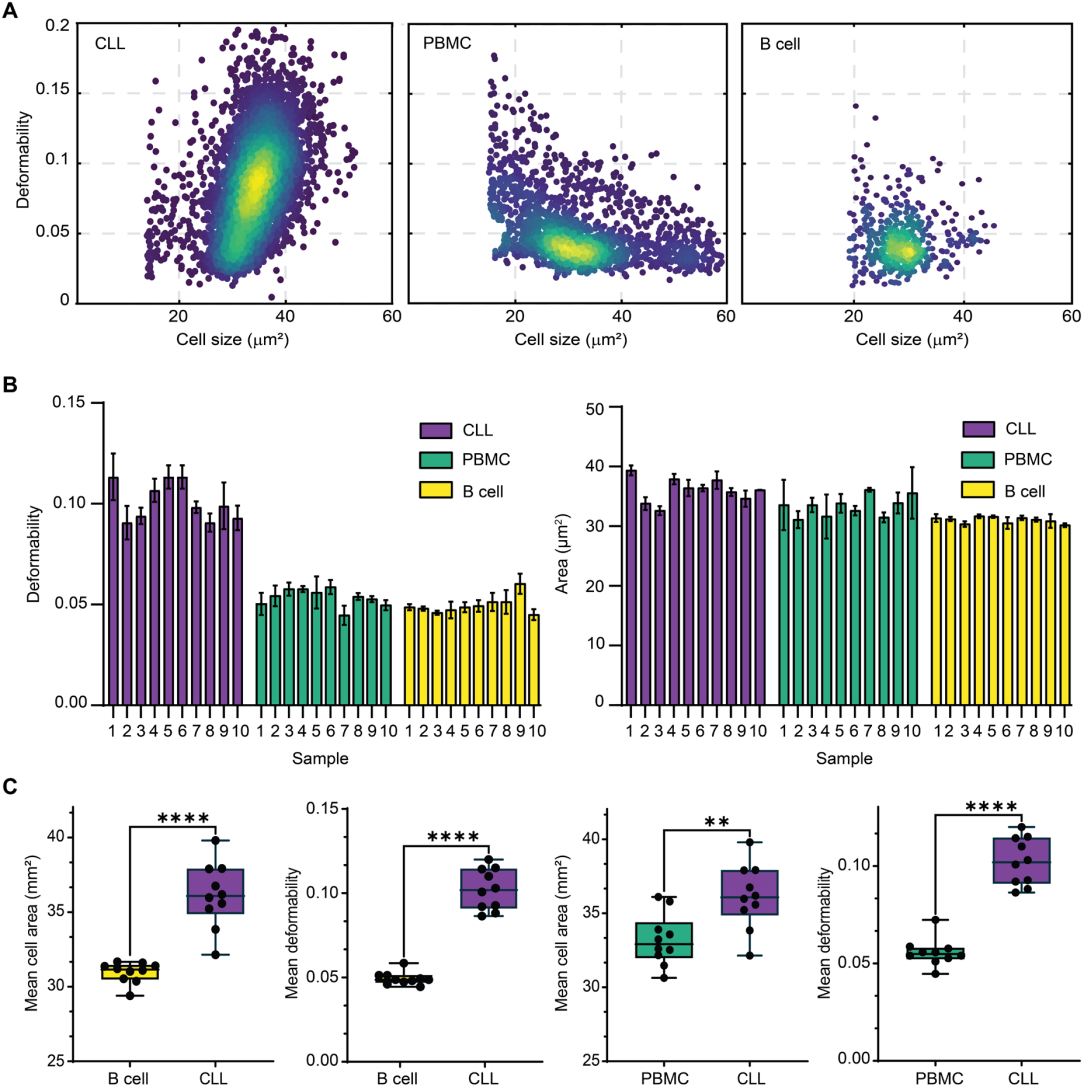
Investigation of CLL mechanical phenotyping using viscoelastic deformability cytometry. (**A**) Deformability versus cell size density scatterplots for samples containing CLL, healthy PBMCs, and healthy B cells. (**B**) Comparison of deformability and size for 10 healthy PBMC, 10 healthy B cell, and 10 CLL samples. For each sample, at least three replicates were measured, each encompassing 5000 cells. (**C**) Box graphs of mean cell deformability and size of control (green) and leukemia samples (purple). The horizontal line represents the phenotype mean values. Box plot whiskers range from the 5th to 95th percentiles and the length of the whiskers is restricted to a maximum of 1.5 times the interquartile range. Statistical comparisons between B cells, PBMCs, and CLLs were performed using a two-tailed Mann-Whitney *U* test; B cells versus CLLs have a significant mean cell size difference (*P* < 0.0001) and deformability (*P* < 0.0001); PBMCs versus CLLs have a significant mean cell size difference (*P* = 0.0089) and deformability (*P* < 0.0001). CLL samples show significantly higher mean deformability and mean size values than the control (healthy) B cell and PBMC samples. A minimum of 5000 cells were imaged in each experiment. On the basis of a camera frame rate of 1000 frames/s, experiments were completed within 500 ms.

As already shown, mechanical phenotyping through the measurement of deformability represents a potentially powerful diagnostic tool. Because cells are known to undergo physical and biological changes during cancer progression (*57*), a complete understanding of such changes offers a route to developing new diagnostic and prognostic tools. Leukemia is a malignant disease of the hematopoietic system, with survival rates ranging from 90% for “good-risk” disease to 20% for “poor-risk” disease (*58*). Contemporary leukemia diagnostics is typically based on a combination of conventional microscopy, flow cytometric analysis of fluorescent cell surface markers, and genetic analysis (*59*). Such diagnostic workflows are complex, costly, and time-consuming. Accordingly, the development of label-free, cost-effective, and rapid diagnostics would transform both the manner in which hematological malignancies are identified and treatment outcomes (*60*). It should be noted that deformability cytometry has already been used as a label-free diagnostic for malignant pleural effusions, including the discrimination of leukemia from inflammatory processes (*5*). However, to date, no study has demonstrated the use of mechanical phenotyping to characterize leukemia cells in a clinical scenario. To address this gap, we used vDC to measure and compare the deformability of isolated CLL cells and healthy lymphocytes from both healthy and leukemia-affected (human) blood samples. We aimed to classify and mechanically characterize CLL cells obtained from patients with lymphoma and compare them with PBMCs and B cells from healthy individuals. Figure 6A presents scatterplots of deformability versus cell size for malignant CLL cells, healthy PBMCs, and healthy B cells, highlighting the power of both size and deformability information in discriminating cell type. Additional analyses performed on cells obtained from a cohort of “healthy donors” and “patients with leukemia” demonstrates considerable differences in both cell size and deformability. Specifically, 10 PBMS samples and 10 B cell samples from healthy donors and 10 CLL samples were analyzed using vDC. Figure 6B (left) shows that CLL cells are significantly more deformable than healthy PBMC and B cells, indicating a potential diagnostic utility of deformability as a CLL reporter. Statistical *t* test analyses confirmed that the mean cell deformability of CLL cells is 0.09, a value that is significantly higher (*P* < 0.0001) than that for PBMCs and B cells, with mean values close to 0.05 (Fig. 6C). In addition, complete analysis of all samples revealed that CLL cells had a higher mean cell area than healthy PBMCs (*P* < 0.0298) and B cells (*P* < 0.0001) (Fig. 6C). These data indicate that CLL cells have a significantly higher mean cell area compared to PBMCs (*P* < 0.0298) and B cells (*P* < 0.0001), suggesting that size differences alone can potentially be used to distinguish different patient types. However, the SD overlap between CLL cells and healthy PBMCs indicates limitations in using size alone as a robust phenotype signature for the characterization of different blood cells. Our findings demonstrate statistically significant differences in deformability, indicating the potential utility of these measurements in distinguishing between cell types within diagnostic settings.

## DISCUSSION

We have developed and characterized a deformability cytometer able to measure the mechanical properties of single cells at exceptionally high throughput and in a label-free manner. Cell deformation is achieved by leveraging both microfluidic confinement and fluid viscoelasticity, with cells being aligned and deformed in a single device and without the need for any sheath fluid. To assess the utility of the vDC platform in clinical scenarios, we performed cell phenotyping of both liquid and solid tumor biopsies, analyzed the impact of cytoskeletal drugs on cell mechanics, and characterized the mechanical properties of malignant lymphocytes isolated from peripheral blood samples.

We have used the vDC platform to identify potential diagnostic signatures of various diseases using a range of bodily fluid and tissue samples. Such an approach circumvents many of the challenges associated with identifying and detecting molecular diagnostic markers and, therefore, should be applicable to a wide range of hematological malignancies. For example, altered cellular deformability is a key component of multiple red cell abnormalities, such as sickle cell disease or thalassemia (*61*–*63*). In this regard, vDC is likely to be highly useful in the diagnosis of hereditary hemolytic anemias (*64*). Currently, osmotic gradient ektacytometry is recognized to be the gold standard in hemolytic anemia screening. Unfortunately, ektacytometry is technically demanding and only established in a small number of hematological laboratories worldwide. Deformability measurements using the vDC platform could provide useful diagnostic information in a cost-effective and rapid manner, while requiring only minute amounts of whole blood as an input. In addition, the simplicity of the vDC (with respect to both fluidics and imaging) ensures that the platform can be easily and widely implemented in hospitals and clinics, rather than in centralized testing facilities, as is currently the norm for flow cytometry instrumentation. Last, automated detection of PBMC cells offers an interesting approach for the potential diagnosis of leukemia, while concurrently excluding clinically similar inflammatory diseases and infections (*65*).

Single-cell mechanical measurements have the potential to significantly advance personalized medicine by providing insights into how individual cells, particularly within tumors, respond to treatment (*66*). For example, tumors comprise heterogeneous cellular subpopulations, with not all cells responding uniformly to a specific therapy. Drugs such as paclitaxel, doxorubicin, and aurora-kinase inhibitors target specific cellular structures and processes, often altering the mechanical properties of cells. By measuring these properties before and after treatment, clinicians could identify resistant cells that may not be detectable through genetic screening alone. Such measurements would provide functional insights into drug efficacy and resistance mechanisms. As a result, the measurement of cell mechanics could be particularly useful for early disease detection, allowing for better patient-specific treatments and improved disease monitoring. Nonetheless, converting these experimental insights into practical therapeutic strategies is not straightforward due to potential off-target effects that can arise from specific proteins, genes, or molecular structures.

Perhaps the most seductive feature of the vDC platform is its high-throughput operation. Deformability can be measured in real time at rates up to 10,000 cells/s, a value that is two orders of magnitude higher than the current gold standard (*41*). Analytical throughput is a critical issue in many clinical scenarios, where many millions (or even billions) of cells must often be analyzed to detect and identify rare cells (such as CTCs) (*67*). The microfluidic device used in our experiments consists of 10 parallel channels, which enables multiplexing and simultaneous measurement of large numbers of individual cells. This configuration enhances throughput and allows for efficient analysis of cell behavior. To mitigate the potential issue of channel blockage, we implemented a filtration region at the inlet of the microfluidic channels. This setup effectively prevents doublets and cell clumps from entering the channels, thereby minimizing the risk of channel blockage during sample injection. In addition, we maintained sample concentrations below 10^7^/ml to further reduce the likelihood of blockages caused by cell aggregates.

In this regard, vDC represents a paradigm shift in the application of flow cytometry to hematological diseases and solid tumor mechanical phenotyping. As noted, and primarily due to the simplicity of the platform, we expect that clinical application of vDC will be both straightforward and immediate, with clinicians adopting deformability as a powerful phenotype in their clinical workflows. In operational terms, our experimental setup employs a pressure pump to inject the sample into the microfluidic device. Use of a pressure pump ensures that a constant pressure differential between the inlet and outlet is maintained, even if one or more channels become blocked. This capability ensures that the fluid velocity in the unblocked channels remains consistent with that of the unclogged system. In regard to acquisition speed and data analysis, the primary limiting factor is typically the camera frame rate and the computational requirements of real-time image processing. Our setup is optimized to capture and analyze 10,000 cells/s in real time, enabling rapid characterization of cell behaviors within the microchannels.

More generally, vDC has the potential to transform the clinical assessment of blood disorders, because diagnostic information is generated in an ultrafast and automated fashion. Last, because the physical properties of cells (such as deformability, shape, and size) are label-free biomarkers, the development of tools for their rapid and precise measurement will undoubtedly engender notable advances in personalized medicine.

## MATERIALS AND METHODS

### Microfluidic device fabrication

Microfluidic devices were fabricated using conventional and soft lithographic techniques. The fabrication workflow is summarized in fig. S18. The microfluidic device used for all experiments comprises three functional sections: a focusing zone, a deformation zone, and a relaxation zone. The focusing zone consists of 10 parallel channels that are 30 mm long, 50 μm wide, and 50 μm high. The deformation zone comprises 10 parallel channels that are 300 μm long, and the relaxation zone comprises 10 parallel channels that are 300 μm long, 50 μm wide, and 50 μm high. The width and height of the straight microchannel in the deformation region are optimized to accommodate cells having 1.5 to 2 times the diameter of the cells being investigated. This ratio ensures that cells undergo the desired deformation as they pass through the microchannel. Furthermore, microchannel dimensions are adjusted based on the size of the cells being analyzed. For instance, in the case of blood cells and mammalian cell lines, constriction microchannels have 15 μm by 15 μm cross sections, while for the larger glioma cells, microchannels have 28 μm by 28 μm cross sections. In addition, a filter region consists of an array of microcolumns. These microcolumns have a diameter of 30 μm, are spaced 30 μm apart, and have a lateral shift of 15 μm between each line. This arrangement acts to filter out cell aggregates, while allowing a maximum of two cells to pass between the microcolumns at any time.

Because the microfluidic channels in the deformation zone are shallower than channels in the focusing and relaxation zones, the soft-lithography master mold was fabricated in two steps using standard photolithographic methods. Briefly, two mask patterns were designed in AutoCAD 2019 (Autodesk, San Francisco, USA) and printed onto 177-μm-thick fine-grain emulsion films to form photomasks (fig. S19) (Micro Lithography Services Ltd., Chelmsford, UK). The first mask was used to define the dimensions of the focusing and relaxation zones, with the second mask determining the dimensions of the deformation zone. The first layer of the master mold was fabricated as follows: SU-8 2010 photoresist (Micro Resist Technology, Berlin, Germany) was spin coated (at 1500 rpm for 30 s) onto a 4-inch (10.16-cm)–diameter silicon wafer and prebaked at 65°C for 3 min and 95°C for 9 min. Next, the photoresist layer was exposed to UV radiation (150 mJ/cm^2^) through the first mask and postbaked at 65°C for 2 min and 95°C for 4 min. The photoresist was then developed for 3 min and hard baked at 150°C for 10 min. This resulted in an initial master mold with feature heights of 15 μm (fig. S18A). The second layer of the master mold was fabricated as follows: SU-8 2050 photoresist was spin coated (at 3000 rpm for 30 s) onto the first layer and prebaked at 65°C for 3 min and 95°C for 9 min. Subsequently, the photoresist was exposed to UV radiation (150 mJ/cm^2^) using the second mask and mask aligner, and postbaked at 65°C for 2 min and 95°C for 4 min. The photoresist was then developed for 5 min and hard baked at 150°C for 10 min. This second layer contains 50-μm high features and is located adjacent to the first layer (fig. S18B). To fabricate microfluidic devices, a 10:1 mixture of polydimethylsiloxane (PDMS) monomer and Sylgard 184 curing agent (Dow Corning, Michigan, USA) was poured over the master mold and polymerized at 70°C for 4 hours (fig. S18C). After curing, the fluidic channel layer was peeled from the support wafer. Individual devices were diced using a scalpel and inlet and outlet ports formed using a hole-puncher (SYNEO, West Palm Beach, USA) (fig. S18D). The structured PDMS substrate was then bonded to a 1-mm-thick glass slide after exposing all internal surfaces to an oxygen plasma (EMITECH K1000X, Quorum Technologies, Lewes, UK) for 60 s (fig. S18E). A schematic of the complete device is shown in fig. S18F.

### Cell culture and sample preparation

Jurkat (Sigma-Aldrich, Buchs, Switzerland), BT474 (ATCC, Manassas, USA), and MDA-MB468 (ATCC, Manassas, USA) cell lines were cultured in polystyrene flasks using Gibco high-glucose Dulbecco’s modified Eagle’s medium (Thermo Fisher Scientific, Lengnau, Switzerland) and Gibco RPMI 1640 (Thermo Fisher Scientific, Lengnau, Switzerland) supplemented with 10% (v/v) fetal bovine serum (FBS) (Thermo Fisher Scientific, Lengnau, Switzerland) and 1% (v/v) penicillin-streptomycin (Thermo Fisher Scientific, Lengnau, Switzerland). LTC (LN-428, LN-18, LN-229, and T98G) and GIC (ZH-161, ZH-562, ZH-305, GS9, and GS5) glioma cell lines were cultured as described previously (*68*). Culture flasks were stored in a Galaxy 170S incubator (Eppendorf, Schönenbuch, Switzerland) at 37°C, 5% CO_2_ and 95% humidity, with media being refreshed every 48 hours. For all experiments, cellular concentrations were maintained at approximately 2 million cells/ml. Before each experiment, cells were centrifuged (120 rpm for 5 min at 24°C), washed in PBS, filtered through a 40-μm pore size strainer (Corning, Corning, USA), centrifuged, and resuspended in PBS. Cell viability experiments before and after deformability measurements were performed using the Zombie viability kit (BioLegend, San Diego, USA). Modification of cell stiffness was achieved via cell fixation and administration of Lat B, Cyto D, and Noco all purchased from Sigma-Aldrich (Buchs, Switzerland). Cyto D, Lat B, and Noco were dissolved in dimethyl sulfoxide (DMSO) according to standard protocols (*21*, *41*, *42*). The following concentrations were used for dose-response experiments: 0.01, 0.1, 1, and 10 μM Cyto D; 0.1, 1, 10, and 100 μM Noco; and 25, 50, 125, and 250 nM Lat B. A 0.1% DMSO solution was used in all experiments to minimize effects on cell deformation. Fixation was performed by the addition of a 4% paraformaldehyde solution for 10 min at room temperature. To prevent the formation of cell aggregates, cells were subsequently washed with PBS supplemented with 10% FBS. PEO (1, 2, and 5 MDa; Sigma-Aldrich, Buchs, Switzerland) was dissolved in PBS to a concentration of 1% (w/v) to create viscoelastic solutions. These solutions were then aged for 1 week at 4°C to ensure steady-state viscosities. Before the experiment, PEO solutions were added to cell media at concentrations of 0.1, 0.5, and 0.8%. The osmolalities of buffers containing varying concentrations of PEO and different molecular weights were measured using the OSMOMAT 3000 instrument (Gonotec, Berlin, Germany). The viscosities of PEO solutions at different shear rates were measured using an MCR 502 compact rheometer equipped with a double gap (DG 26.7) tool (Anton Paar, Ostfildern, Germany).

### Human blood samples

All blood samples were collected in EDTA tubes. CLL samples from patients with cancer (10 in total) were obtained from the University Hospital Zurich (Zurich, Switzerland). Blood samples from healthy donors (10 in total) were obtained from the blood donation center, Blutspende Zürich (Schlieren, Switzerland). The study was conducted in accordance with the principles of the Declaration of Helsinki, and both projects (related to HD and CLL samples) were approved by the Swiss Association of Research Ethics Committees (BASEC-Nr: 2019-01721, BASEC-Nr: 2019–01744, and KEK-Nr: 2009-0062/1).

### PBMC isolation

Mononuclear cells from human whole blood of healthy individuals and patients with CLL were isolated by density centrifugation using SepMate-50 tubes (STEMCELL Technologies, Vancouver, Canada). Briefly, a density gradient medium (1.077 g/ml) was added to the tubes through the insert, and blood samples, previously diluted at a ratio of 1:2 with PBS and supplemented with 2% FBS, were added. Density centrifugation was performed at 1200*g* for 10 min at room temperature. Subsequently, the top fraction (containing enriched mononuclear cells) was removed, washed twice with PBS at 300*g* for 8 min, and lastly resuspended in PBS.

### Deformability cytometry

A Flow EZ pressure pump (Fluigent, Paris, France) was used to motivate cells through the microfluidic device. High-speed bright-field imaging of cells was performed using a CB019MG-LX-X8G3 high-speed camera (XIMEA, Münster, Germany) mounted onto a Ti-E microscope (Nikon, Zürich, Switzerland) equipped with a 20×, 0.45 NA (numerical aperture) S Plan Fluor objective (Nikon, Zürich, Switzerland) and a collimated UHP-T-560-DI-DF LED (Prizmatix, Holon, Israel) source. For an ROI of 1024 × 80 pixels, images were recorded at 10,000 frames/s. In addition, to ensure the acquisition of blur-free images, the camera was operated using an exposure time of 1 or 2 μs. Images captured by the high-speed camera were transferred to a Linux multicore PC using a camera-link frame grabber card.

### Image analysis

All image processing steps were performed using a Linux multicore PC running custom-written software. Image processing algorithms were implemented in Python, and for real-time operation, an algorithm capable of performing image acquisition, image analysis, and data storage for several hundred cells per second was developed using the OpenCV computer vision library (http://opencv.org).

Image processing consists of the following steps: (i) background subtraction, (ii) threshold filtering, (iii) contour finding, and (iv) contour processing to allow estimation of cell size, deformation, and brightness. For a selected ROI, the video is cropped, and a background image is calculated by averaging the intensities of the captured images. A median filter is used to reduce the noise and a binary filter (with a specified threshold) is used to identify cell contours. Subsequently, the area, *A*, and perimeter, *l*, of each detected cell within the image is determined. The values are then used to calculate the deformability, *D*.

Last, a two-dimensional scatterplot of deformability versus cell area is created, with a single contour line defined at 50% density. It should also be noted that other physical parameters can be extracted from images in real time. These include brightness and aspect ratio. Brightness is defined as the mean of all pixel intensities inside a given cell contour, and aspect ratio is defined as the ratio of the large to the small axis of a given cell image. Last, the ratio of the convex hull area to the measured cell area (the area ratio) is calculated and used to indicate the presence of a cell (fig. S20).

The software architecture has a multithread structure, with communication between different threads being achieved through queues. Threads receive data packets and calculate all parameters in real time. When a cell passes through the constriction channel, the camera measures and streams the video frame to the software threads. In practice, the first thread is responsible for acquiring single frames from the camera and depositing them into a circular buffer. The second thread performs the image processing tasks, including background subtraction, thresholding to create a binary image, cell contour detection, and calculation of the cellular attributes. Processed data packets including scatterplots and images of the detected cell contours are then sent to the Graphical User Interface (GUI) for display and continuously updated using the third thread. The GUI handles user inputs and configuration, such as selecting an ROI. Scatterplots of cell size versus deformability (generated in real time) are displayed in the GUI (fig. S21) and stored together with results from image processing (including brightness, aspect ratio, and an image of the cell) for subsequent analysis. The processing time (of a single frame) for extracting all these parameters is between 0.5 and 1 ms.

### Statistical analysis

All statistical analyses were performed using Prism 9.4 (GraphPad, La Jolla, USA). In all analyses, the significance level is defined as *P* < 0.033, indicated by *; *P* < 0.0021, by **; *P* < 0.0002, by ***; and *P* < 0.0001, by ****. The statistical significance of overall differences of cell size and deformability data derived from the drug-treated and glioma cell experiments was analyzed by one-way analysis of variance (ANOVA) followed by Tukey’s multiple comparison test. CLL cell, B cell, and PBMC deformability and cell size data were analyzed using a two-tailed Mann-Whitney U test. Such a *t* test was used to assess unpaired samples of healthy individuals versus patients with blood cancer. The *P* values reported from each experiment were generated through comparison of the given sample to the control condition.

### Numerical simulations

CFD simulations were used to evaluate the distribution of hemodynamic shear stress on the cell surface due to fluid viscoelasticity (fig. S22). A representative geometrical model of a cell (10 μm in diameter) moving through a deformation channel (15 μm wide and 15 μm high) was created for this purpose. Following a prior mesh independence study, the geometry was discretized into ~1.05 million polyhedral and prismatic (five layers) elements. Steady-state, laminar simulations of the four PEO concentration conditions were performed using v. 2020 Ansys Fluent (Ansys, Canonsburg, USA). The boundary conditions were derived from the experimental setup of the considered scenario. Specifically, the mean velocity (imposed as a flat profile) at the inlet was derived from relative flow-rate measurements at the inlet of the microfluidic device, assuming quantity conservation. At the outlet, a relative pressure of zero was assumed. The no-slip condition was imposed on the rigid walls of the microfluidic channel and the cell. Preliminary comparisons of the model were performed by comparing Newtonian and non-Newtonian Carreau models and assuming a constant density of 1080 kg/m^3^ (fig. S22). For each PEO concentration, rheological data were extracted and introduced into Prism 9.4 to derive Carreau model parameters. All additional simulation settings are summarized in table S3.

## Supplementary Materials

### The PDF file includes:

Texts S1 to S8 Figs. S1 to S22 Tables S1 to S3 Legend for movie S1 References
