# Supplementary material for: Real-time viscoelastic deformability cytometry: High-throughput mechanical phenotyping of liquid and solid biopsies
Source: Sci Adv. 2024 Dec 4;10(49):eabj1133. doi: 10.1126/sciadv.abj1133 (PMC11616701; doi:10.1126/sciadv.abj1133)
Supplement: Supplementary file 1 — Texts S1 to S8 Figs. S1 to S22 Tables S1 to S3 Legend for movie S1 References [file sciadv.abj1133_sm.pdf]

Science Advances
AAAS

Supplementary Materials for

Real-time viscoelastic deformability cytometry: High-throughput mechanical phenotyping of liquid and solid biopsies

Mohammad Asghari *et al.*

Corresponding author: Stavros Stavrakis, stavros.stavrakis@chem.ethz.ch;
Andew J. deMello, andrew.demello@chem.ethz.ch

Sci. Adv. **10**, eabj1133 (2024)
DOI: 10.1126/sciadv.abj1133

The PDF file includes:

Texts S1 to S8
Figs. S1 to S22
Tables S1 to S3
Legend for movie S1
References

Other Supplementary Material for this manuscript includes the following:

Movie S1

Supplementary Text 1. Particle focusing in elasto-inertial systems.

Passive manipulation of particles/cells within microfluidic systems can be achieved by exploiting the flow conditions and fluid properties. The dimensionless Reynolds number, Re, characterizes the flow condition and is defined as the ratio of inertial to viscous forces in a medium:

$$Re = \frac{\rho U D_h}{\eta} \quad (1)$$

where, ρ is the fluid density, U is the fluid velocity, and D_h is the hydraulic diameter, defined as $D_h = \frac{2wh}{h+w}$, where h and w are the height and width of the channel, respectively. In a Poiseuille flow, when the inertia effect is not negligible (Re >10), a considerable inertial lift force is generated, which causes lateral motion of suspended particles due to the velocity gradient. Hood *et al.* (*69, 70*) recently derived a general equation for inertial lift force, F_{iL},:

$$F_{iL} \sim \frac{\rho U^2 r^4}{h^2}\left[c_4 + \frac{r}{h} c_5\right] \quad (2)$$

Here, U is the average velocity, r is the particle radius, and c_4 and c_5 are the asymptotic expansion coefficients, which depend on the particle location and the rectangular channel's aspect ratio. The polymeric suspension in viscoelastic solutions introduces an elastic behavior to the fluid altering its response to flow conditions. The dimensionless Weissenberg number, Wi, describes the relation between elastic and viscous forces within such a fluid:

$$Wi = \lambda\dot{\gamma} = \frac{2\lambda Q}{hw^2} = \lambda\frac{2U}{w} \quad (3)$$

where, λ is the relaxation time of the fluid, $\dot{\gamma}$ is the characteristic shear rate, and Q is the flow rate. Unlike the constant viscosity in Newtonian fluids (η, only dependent on temperature), the viscosity of viscoelastic fluids is typically a decreasing function of the shear rate due to the macromolecular nature. This phenomenon is known as shear thinning and is found to drive outward migration of particles in a viscoelastic flow. The elastic behavior in these fluids is related to the normal stresses generated by the orientation and alignment of macromolecules along the flow direction (*71*). The difference between normal stresses, N_1 (difference between axial and lateral directions) and N_2 (difference between lateral and angular directions) causes particle cross-stream migration in a viscoelastic flow. Generally, N_2 is much smaller than N_1 and is therefore often neglected. Accordingly, the elastic force can be expressed as:

$$F_{eL} \sim a^3 \nabla N_1 \sim \lambda_e \left(\frac{a}{w}\right)^3 Q^3 \quad (4)$$

where, a is the spherical diameter of the particle, and λ_e is the effective relaxation time of the fluid (*72*). The combination of both inertial (eq. 2) and elastic (eq. 4) forces results in the central positioning of particles within a square microchannel cross-section. We performed a numerical simulation using COMSOL Multiphysics 6.0 to evaluate the inertial and elastic force fields in a cross section of a square channel. **Fig. S1** shows an example of simulation results using a flow rate of 0.04 µL/s in a 50 µm × 50 µm square channel filled with a 1% w/v 1MDa PEO solution. The background color indicates shear rate intensity (blue: minimum value; red: maximum value) across the cross-section. The blue and red arrows represent elastic and inertial forces, respectively. The interplay of these forces determines the particle focusing position. As shown in **Fig. S1 inset**, the force field in a square channel has reflection symmetry, resulting in central particle focusing both vertically and laterally.

Supplementary Text 2: Mathematical model for analyzing cell deformation in PEO viscoelastic fluids.

We simulated the cell deformation process in a viscoelastic medium using the fluid-solid interaction (FSI) model which is implemented in COMSOL Multiphysics 6.0. The fluid was modeled by the incompressible Navier–Stokes equations given by:

$$\rho_f \frac{\partial u}{\partial t} + \rho_f (u.\nabla) u = \nabla. [-pI + \kappa] \quad (5)$$

$$\rho_f \nabla. u = 0 \quad (6)$$

$$\kappa = \mu_f (\nabla u + (\nabla u)^T) \quad (7)$$

where ρ_f is the fluid density, u is the fluid velocity, p is the pressure, I is the identity matrix and μ_f is the dynamic viscosity of the fluid. The Carreau fluid model was used to account for the shear-thinning behaviour of the non-Newtonian PEO solution. Its viscosity is given by the following equation:

$$\mu_f = \mu_{inf} + (\mu_0 - \mu_{inf})[1 + (\lambda \dot{\gamma})^2]^{\frac{n-1}{2}} \quad (8)$$

Here μ_{inf} is the viscosity at infinite shear rate, μ_0 is the viscosity at zero shear rate, λ is the characteristic time, $\dot{\gamma}$ is the shear rate and n is the power index.

Cells were assumed to be incompressible and were represented as non-linear neo-Hookean hyperelastic materials described by the following equation:

$$\rho_c \frac{\partial^2 u_c}{\partial^2 t} = \nabla.(FS)^T \qquad 9$$

Here $\rho_c$is the cell density, u_c is the displacement vector, F is the deformation gradient and S is the second Piola–Kirchhoff stress. The neo-Hookean hyperelastic material is defined by its elastic strain energy density, W_s, i.e.

$$W_s = \frac{1}{2}\mu(\bar{I}_1 - 3) + \frac{1}{2}\kappa(J_{el} - 1)^2 \qquad 10$$

W_s is the sum of the isochoric strain energy density (using the isochoric invariant $\bar{I}_1$) and the volumetric strain energy density (using the elastic volumetric deformation J_{el}). In the case of an incompressible material, the Lamé parameter is $\mu = E/3$, where E is the Young's modulus, and κ is the bulk modulus.

The above system of equations was discretized and solved using the finite element method in COMSOL Multiphysics 6.0. To reduce computational time, symmetric 2D simulations were performed. The computational domain is meshed with a combination of triangular and uniformly distributed mapped meshes. Linear Lagrange elements were used to discretize the Navier–Stokes equations, while linear elements on the cell surface discretize the cell's displacement field.

The inflow profile is pre-computed without a cell, based on the flow rate (velocity), channel width, and non-Newtonian fluid parameters, and applied as a Dirichlet boundary condition at the inlet (*73*). At the outlet, we consider a pressure equal to air pressure, suppressing any potential backflow. To maintain the cell's position at the center of the computational domain, all quantities were described in a coordinate system moving with the cell. The Arbitrary Lagrangian–Eulerian (ALE) method implemented in COMSOL was used to model the deformation of the fluid domain (*74, 75*).

Cell deformation, D, was calculated using the same equation used in the experiments, ie.

$$D = 1 - \frac{2\sqrt{\pi A}}{l} \qquad 11$$

where A is the cell area and l is the cell circumference. Both values were obtained from the simulations.

The simulation was terminated once steady-state deformation was achieved, using an absolute deformation change criterion of $\frac{dD}{dt} \leq 1 \times 10^{-3} s^{-1}$.The fully bidirectional coupled approach was implemented in the fluid-solid interaction (FSI) model. The Newton and the PARDISO solver methods were used for time dependent and spatial discretization parts of Equations 5-10. By solving the system of the above equations, we can derive the shear stresses on the cell and the corresponding cell deformations in relation to the fluid rheology and flow conditions.

Supplementary Text 3: Measurement of sample rheology.

The mathematical model described in **Supplementary Text 2** was used to extract relationships between the deformability, velocity, viscosity, and shear stress in PEO viscoelastic fluids. This mathematical model uses the viscoelastic properties of the 0.1% 1MDa PEO fluid, which were extracted from rheological experiments at different shear rates (**Fig. S8**). **Fig. S8A** reports the shear viscosity of viscoelastic solutions containing PEO of different molecular weight, ranging from 600 kDa to 5 MDa at a concentration of 0.1% w/v. Increasing the molecular weight from 600 kDa to 5 MDa results in an approximately two-fold increase in viscosity. Additionally, **Fig. S8b** demonstrates the impact of PEO concentration on solution viscosity using 1 MDa PEO ranging from 0.05% w/v to 1.0% w/v. Higher concentrations, such as 1.0% w/v, exhibit strong shear-thinning behavior, while lower concentrations show weaker shear-thinning properties. After curve fitting of viscosity plots, in **Fig. S8B** using the Carreau fluid model, we obtained the viscoelastic properties of these PEO solutions, which are summarized in **Table S1.**

Supplementary Text 4. Impact of cell velocity on cell deformability.

Fig. S9 illustrates the impact of cell velocity on its deformation and more specifically how cell shape changes at each flow rate and the corresponding deformability values derived from the simulation. The color bars indicate the velocity magnitude and shear stress distribution around the cell, whilst the streamlines depict the flow patterns around the cell. The simulation parameters are as follows: Young's modulus, E =1 kPa, channel width, w = 15 µm, cell diameter, d = 13 µm and the viscoelastic properties of the 0.1% w/v 1MDa PEO solution derived from curve fitting of rheology measurements (**Fig. S8B**) using the Carreau model (**Table S1**). These values are: μ_0 = 1.55 mPa.s, μ_{inf} = 1.40 mPa.s, λ = 21.22 s, and n =

0.8107. As the cell velocity increases from 0.1 to 0.75 m/s, deformability changes significantly from 0.003 to 0.111 and the cell shape transitions from circular to bullet-like.

Supplementary Text 5. Impact of fluid viscosity on cell deformability.

We analyzed the effect of fluid viscosity on cell deformation by implementing the viscosities of 1 MDa PEO concentrations of 0.1%, 0.5%, 0.8%, and 1.0% w/v (**Fig. S10**). These viscosity values along with the corresponding λ and n values at different PEO concentrations were derived from curve fitting of rheology measurements (**Fig. S8B**) using the Carreau model (**Table S1**). Other parameters used in our simulations were: cell Young's modulus, E = 6 kPa, channel width, w = 15 μm, velocity = 0.35 m/s. As shown in **Fig. S10**, increasing fluid viscosity (from μ = 1.0 mPa.s for Newtonian fluid to μ_{inf} = 18 mPa.s for PEO 1.0%) can result in a two-order magnitude increase in deformability. It should be noted that in the simulations, although varying the PEO concentration can marginally alter cell size due to the effect of osmolarity, we used a constant cell size (13 μm).

Supplementary Text 6. Impact of cell size on cell deformability.

Fig. S11 illustrates the impact of cell size on cell deformability (D). The simulation parameters are as follows: cell Young's modulus E = 1 kPa, flow rate of Q = 0.04 uL/s, channel width w = 15 μm, and viscoelastic parameters for 0.1% w/v 1MDa PEO according to the Carreau model: μ_0 = 1.55 mPa.s, μ_{inf} = 1.40 mPa.s, λ = 21.22 s, and n = 0.8107. The cell size (diameter) ranges from 5 to 14 μm. As the cell size increases from 5 μm to 14 μm, deformability increases from 0.0025 to 0.053.

Supplementary Text 7. Impact of PEO on the osmolality of the buffer.

In addition to influencing cell osmolarity, PEO can also affect the osmolality of the buffer, which in turn can impact the mechanical properties of deformable cells. To evaluate the effect of PEO concentration and molecular weight on buffer osmolality, we measured the osmolality of buffers containing PEO at various concentrations (0.1%, 0.2%, 0.5%, 0.8%, and 1.0%) and molecular weights (1 MDa, 2 MDa, and 5 MDa). As shown in **Fig. S13A**, higher concentrations of PEO lead to a reduction in buffer osmolality. To further investigate this decrease, we measured osmolality at increasing PEO concentrations in DI water instead of PBS. As shown in **Fig**. **S13B**, osmolality increased with higher PEO concentrations. In PBS, the osmolality decrease as PEO concentration is increased (**Fig. S13A**) may be a result of solute agglomeration due to interactions between PEO and buffer components. Furthermore, the increased viscosity (as shown in **Fig. S8**) may also be a contributing factor. Since the OSMOMAT 3000 osmometer used in the current study measures osmolality via freezing point depression, higher viscosities may retard freezing kinetics and/or hinder water crystallization, leading to artificially low osmolality readings. In all cell deformability experiments, we consistently used 1 MDa PEO at a 0.1% w/v concentration (**Fig. S13C**). However, as

shown in **Fig. S12**, this does not affect cell size, and thus our cell deformability studies are not impacted by the presence of PEO.

Supplementary Text 8. Effect of channel clogging on fluid conditions in a pressure-driven system.

To investigate the effect of channel clogging on fluid conditions in a pressure-driven system, we conducted COMSOL simulations. As shown in **Fig. S17A**, a constant pressure of 500 mbar was applied at the inlet for both the ten-channel and single-channel configurations. The resulting flow velocity profiles, shown in **Fig. S17B**, indicate that velocities remain stable and uniform across the channels in both scenarios. This demonstrates that our pressure-driven system effectively maintains consistent fluid velocities even under conditions of channel blockage, offering a significant advantage over a single-channel system.

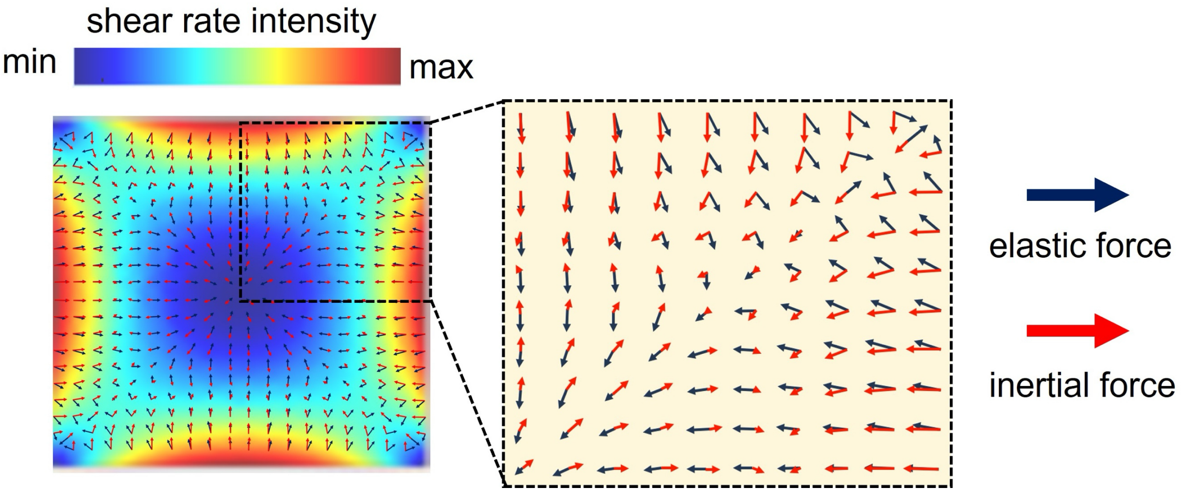

Fig. S1. Illustration of elastic and inertial force arrow-field distributions for a square cross-section microchannel. The color gradient represents the shear rate gradient across the square cross-section channel profile.

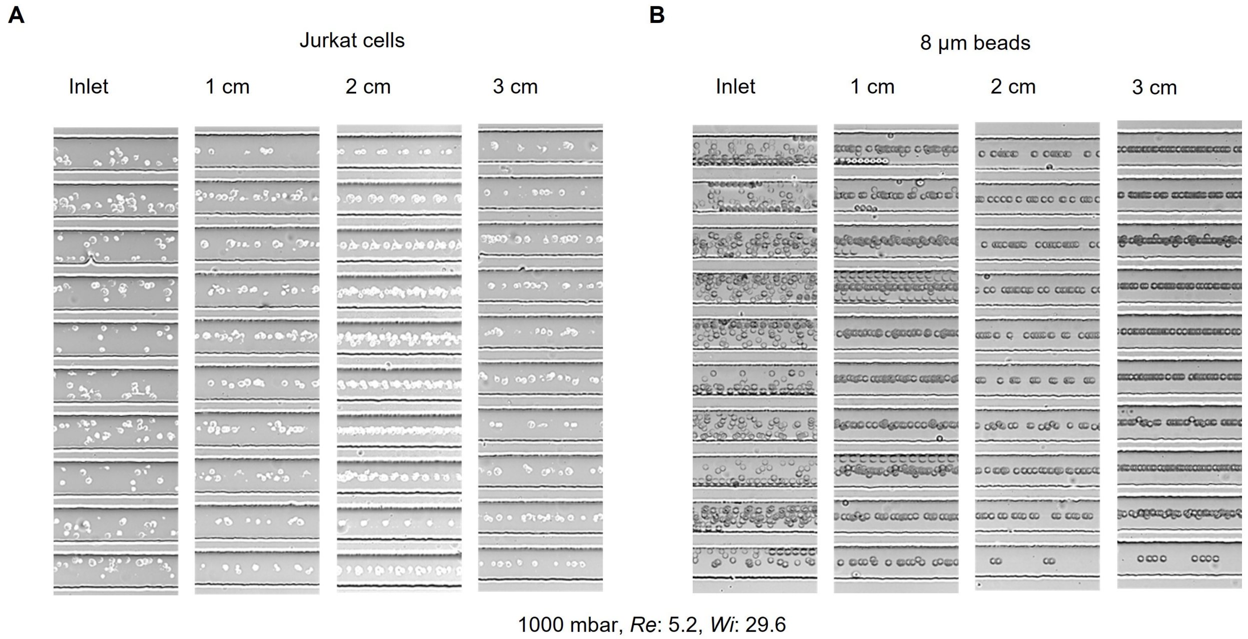

Fig. S2. Focusing performance at various channel lengths. A) Image series demonstrating the focusing of Jurkat cells at increasing channel lengths. B) Image sequence depicting the focusing of 8-micron beads under varying channel lengths.

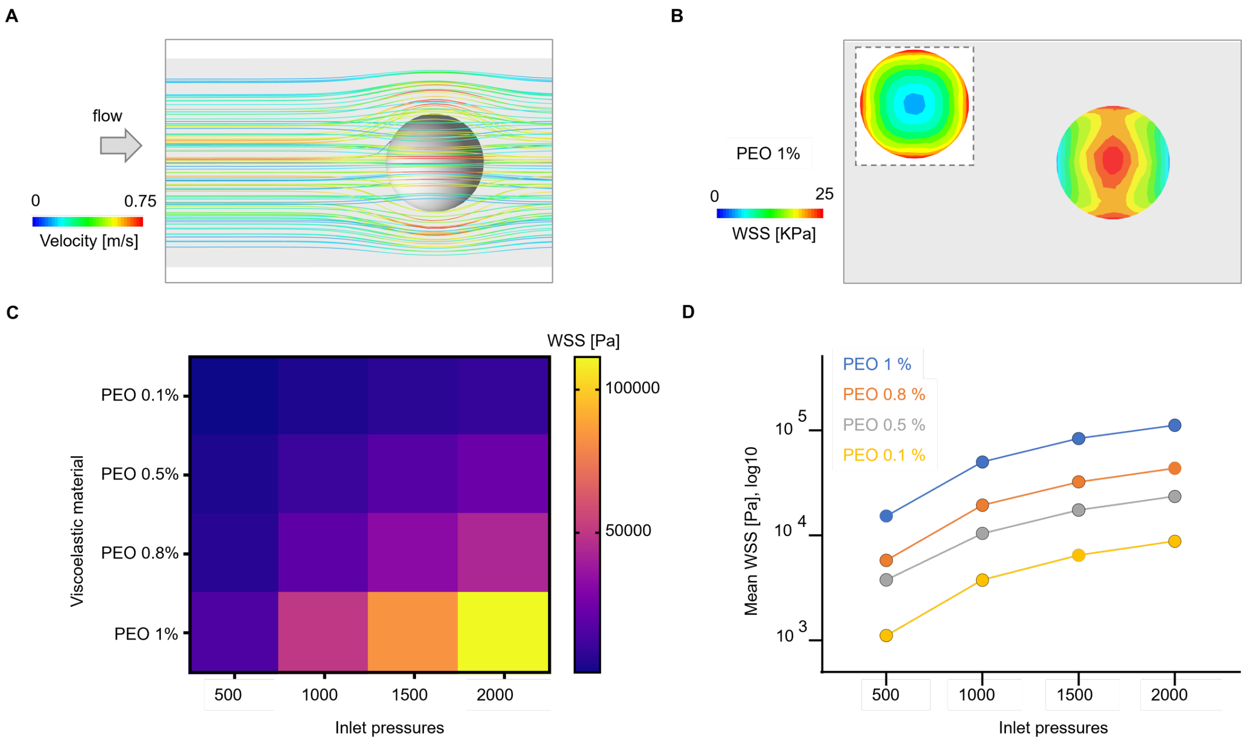

Fig. S3. Impact of the fluid theology and inlet pressure on the cell wall shear stress. A) Flow streamlines around a cell. B) Wall shear stress distribution around a cell. C) Mean WSS versus inlet pressure for PEO 1MDa at concentrations of 0.1%, 0.5%, 0.8%, and 1.0%. D) Plot of mean WSS against cell pressures showing that the mean WSS is increasing when the pressure is elevated.

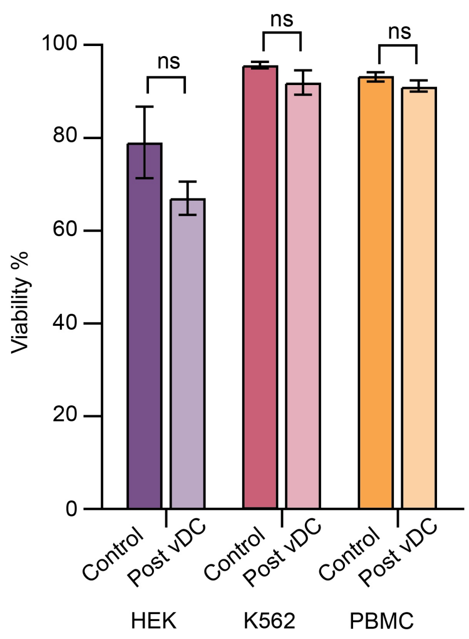

Fig. S4. Viability of different types of cells before and after deformability analysis. The difference in viability of HEK, K562, and PBMC cells before and after deformation is not statistically significant. The height of each bar represents the average of three measurements. Statistical comparisons between the samples were performed using a non-parametric t-test; The mean viability value among the above samples is not significant (ns).

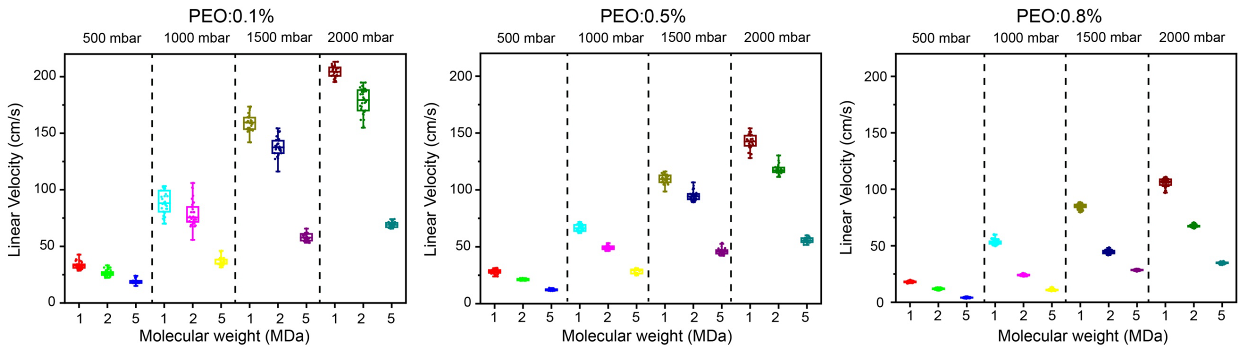

Fig. S5. Variation of the average linear velocity of cells at different inlet pressures and at different PEO concentrations. Comparison of the average linear velocity of cells when suspended in carrier fluids containing three different PEO concentrations (1%, 0.5% and 0.8%) for PEO molecular weights of 1, 2, and 5 MDa and at inlet pressures of 500, 1000, 1500, and 2000 mbar. For each analysis, the velocity of 30 cells was measured at the end of the deformation region.

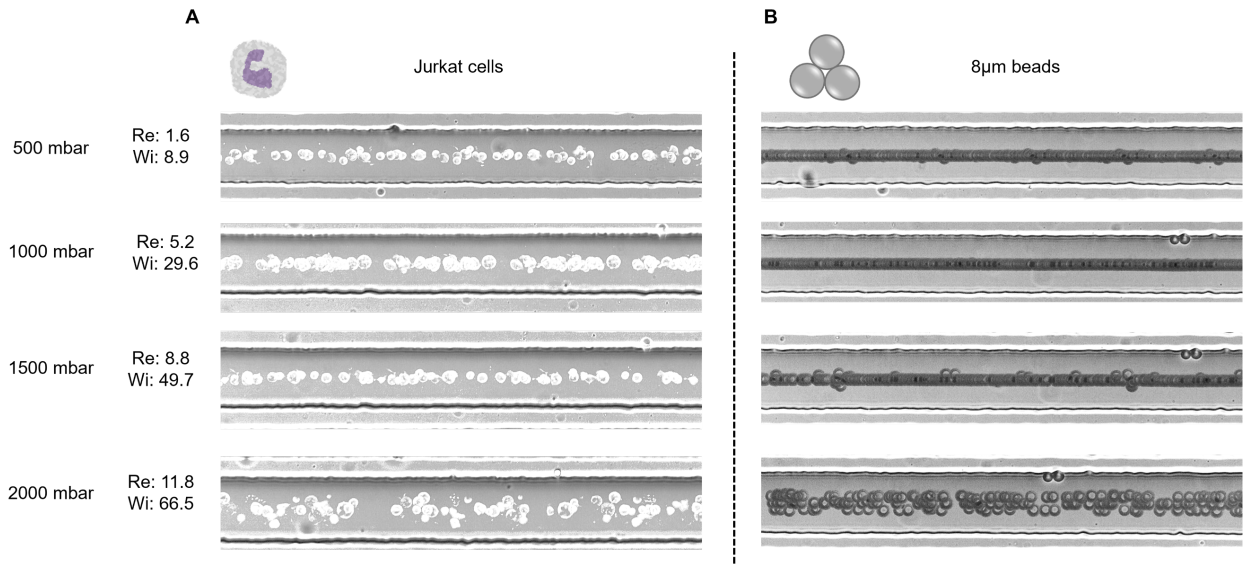

Fig. S6. Focusing performance at various inlet pressures. A) Image series demonstrating the focusing of Jurkat cells at increasing inlet pressures. B) Image sequence depicting the focusing of 8-micron beads under varying inlet pressures

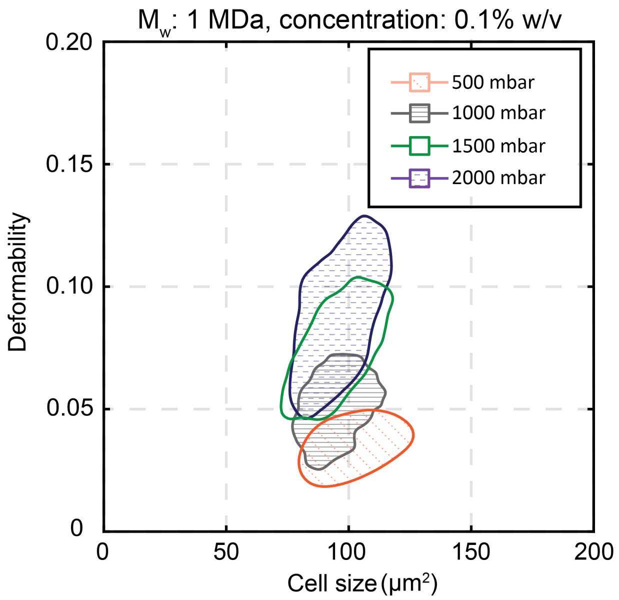

Fig. S7. Scatter plot of deformability vs cell size as a function of inlet pressures. High inlet pressures lead to increased cell deformability.

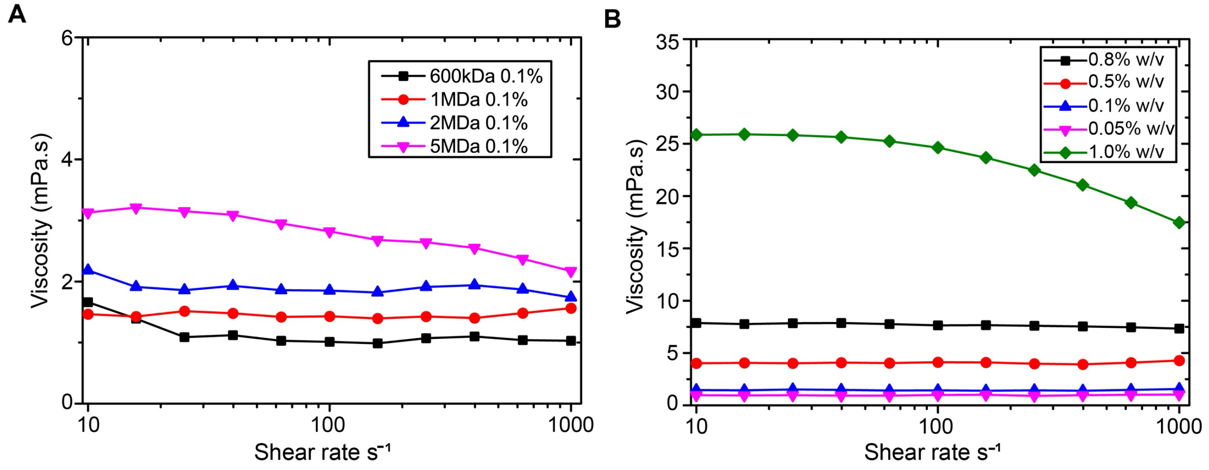

Fig. S8. Measurements of sample rheology. A) Shear viscosity as a function of shear rate for polyethylene oxide (PEO) solutions at 0.1% (w/v) concentration, with molecular weights ranging from 600 kDa to 5 MDa. B) Shear viscosity as a function of shear rate for PEO solutions at 1 MDa molecular weight, with concentrations varying from 0.05% to 1.0% (w/v).

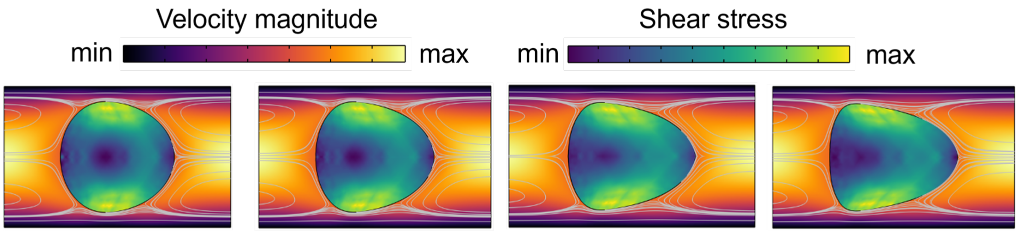

Cell speed (m/s)	0.10	0.18	0.28	0.38
D	0.003	0.012	0.024	0.039

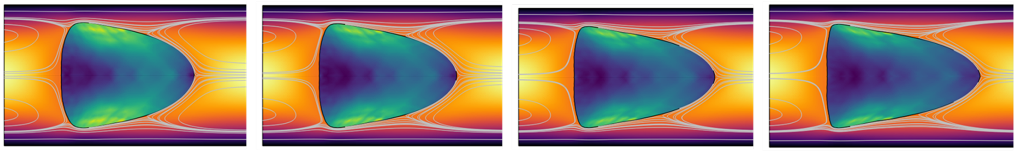

Cell speed (m/s)	0.46	0.56	0.65	0.75
D	0.055	0.072	0.091	0.111

Fig. S9. Effect of velocity on cell deformation and shear stress distribution. The simulations illustrate how the variation of flow rates impacts cell stress distribution, with corresponding deformability values for each flow condition. As the cell velocity increases from 0.1 to 0.75 m/s, deformability changes significantly from 0.003 to 0.111 and the cell shape transitions from circular to bullet-like.

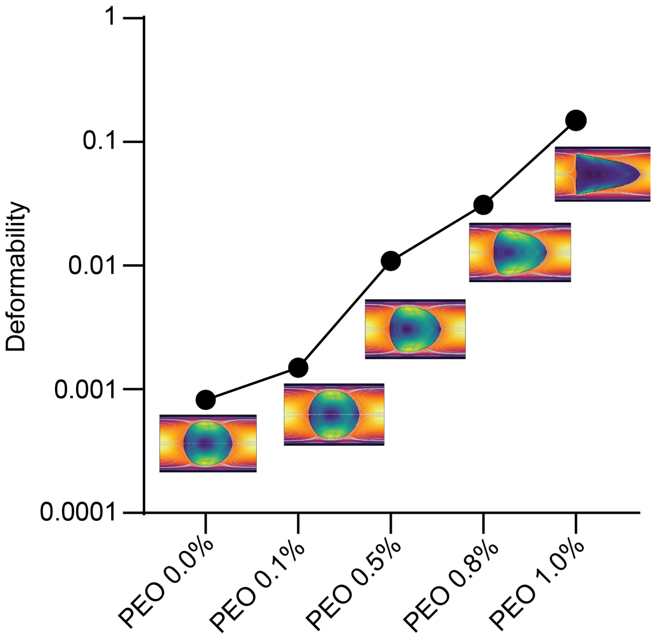

Fig. S10. Impact of fluid viscosity on cell deformation. Cell deformation in Newtonian (PEO 0.0% w/v) and 1MDa PEO solutions at concentrations of 0.1%, 0.5%, 0.8%, and 1.0% w/v. Variations in PEO concentration notably affect cell deformation.

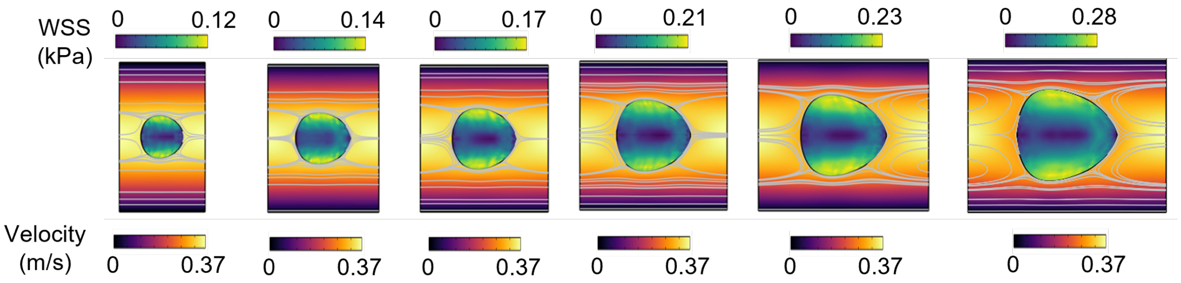

d (µm)	5	6	7	8	9	10
D	0.0025	0.004	0.0055	0.0075	0.010	0.014

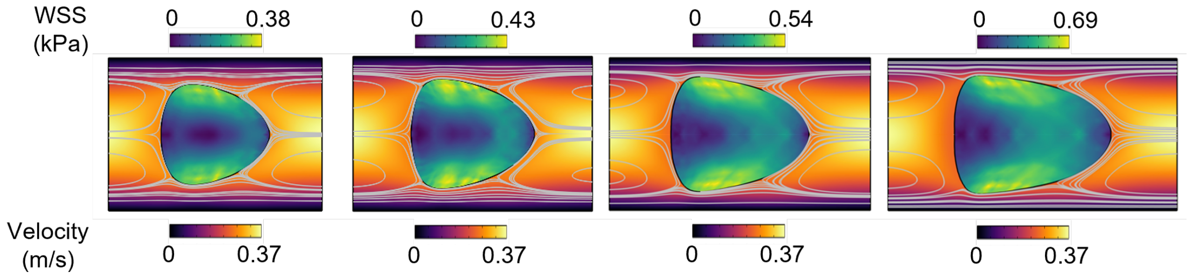

d (µm)	11	12	13	14
D	0.020	0.029	0.039	0.053

Fig. S11. Effect of cell size on cell deformability. Variation cell stress distribution as a function of cell size, with corresponding deformability (D) values for each flow condition. As the average cell size increases from 5 µm to 14 µm, cell shape transitions from circular (D:0.0025) to bullet-like (D:0.053).

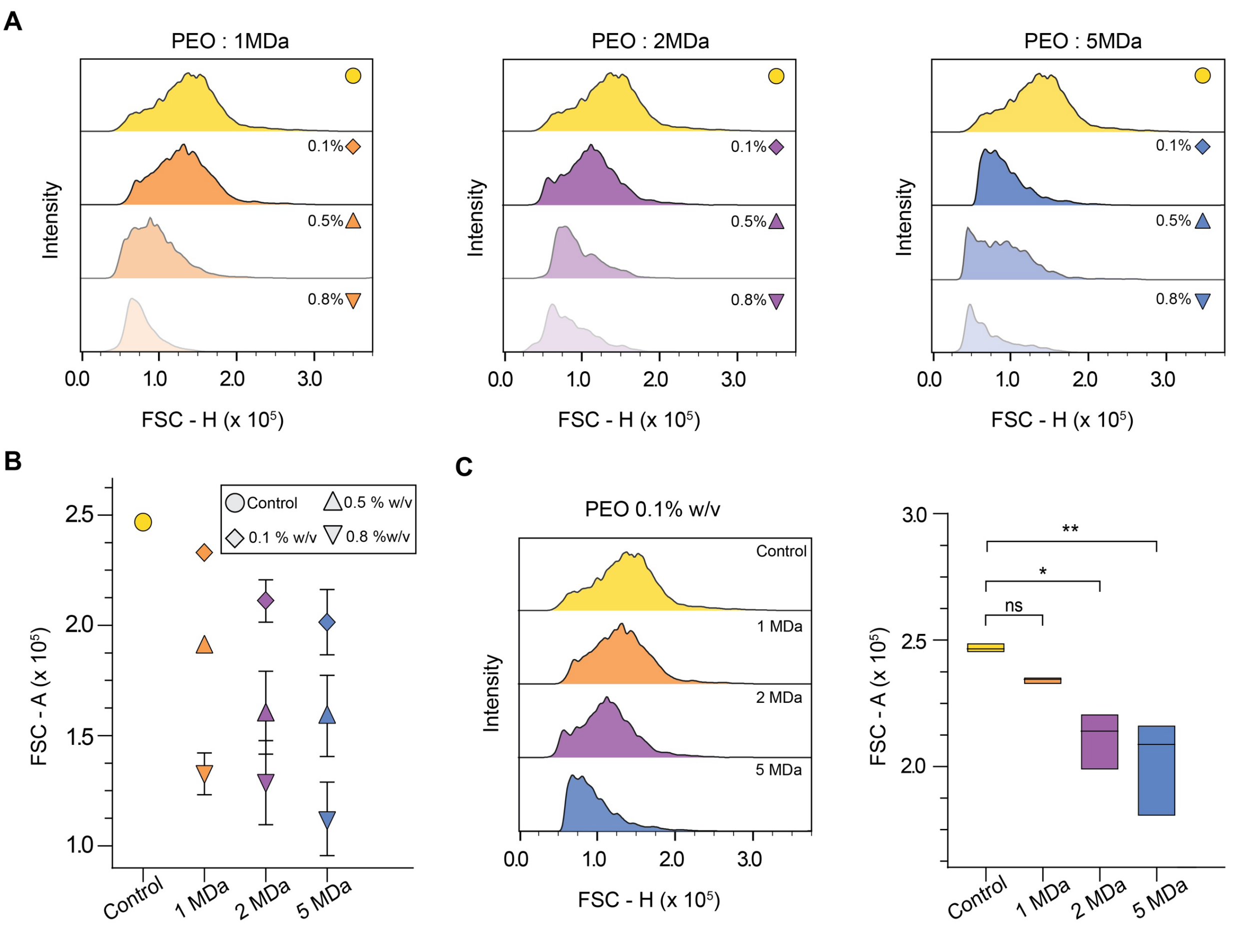

Fig. S12. Analysis of the impact of the molecular weight and concentration of PEO on the size of HEK-293 cells. A) Evolution of cell size for HEK-293 cells at different PEO molecular weights: 1 MDa (orange), 2 MDa (purple), and 5 MDa (blue). Each line represents a different concentration within each molecular weight: 0.1%, 0.5%, and 0.8% w/v. B) Summary of the median FSC values from part (a), with each color representing a different molecular weight and each symbol representing a different concentration. C) FSC values at 0.1% w/v PEO concentration as a function of different PEO molecular weights, showing that the signal difference between the control and 1 MDa is not significant. This demonstrates that the impact of the viscosity of the PEO used in the conducted experiments (0.1% w/v at 1 MDa) is not significant with respect to cell size and does not impact analyses.

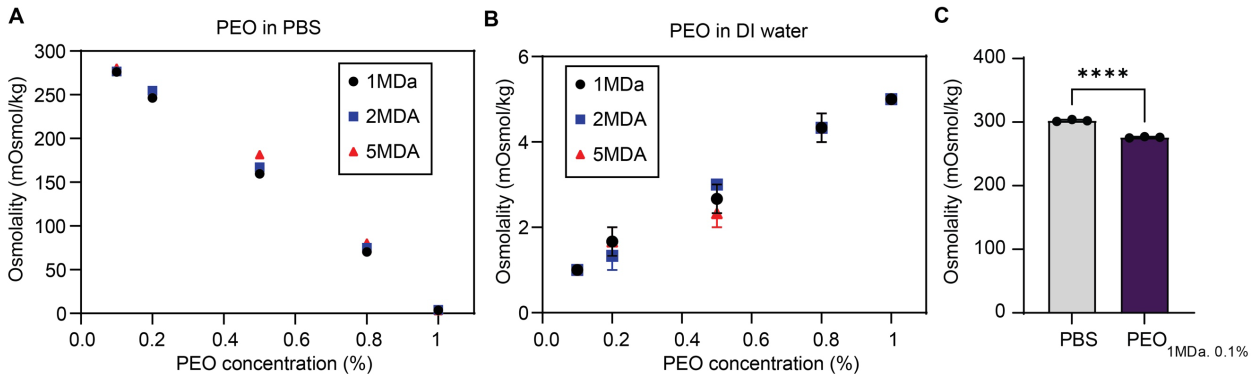

Fig. S13. Impact of PEO molecular weight and concentration on buffer osmolality. A) Osmolality of PBS as a function of PEO molecular weight (1 MDa, 2 MDa, and 5 MDa) and concentration (0.1%, 0.2%, 0.5%, and 1.0% w/v). Increasing PEO concentrations leads to a decrease in osmolality, whilst the variation in molecular weight shows minimal effect. B) Osmolality of DI water as a function of PEO molecular weight (1 MDa, 2 MDa, and 5 MDa) at the same concentrations used in (A). Increasing PEO concentrations lead to an increase in osmolality. C) Addition of 0.1% w/v 1 MDa PEO to PBS causes a measurable change in osmolality.

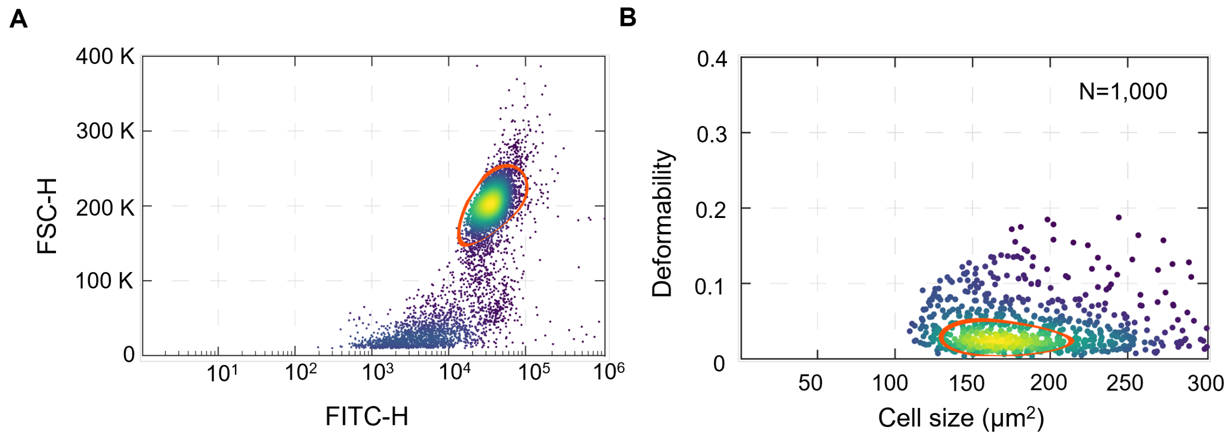

Fig. S14. FC and vDC measurements of LN 229 cells in PBS buffer. To detect the low number of LN229 cells in the FC and VDC spiking experiments, we gated the population of LN229 cells in the 2D density plots of FSC-H vs FITC (A) and deformability vs cell size (B).

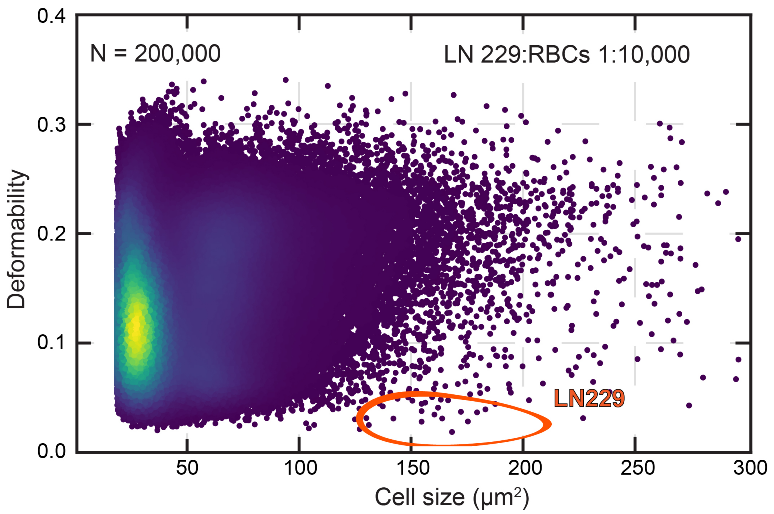

Fig. S15. Rare cell quantification using vDC. LN229 cells were spiked into 10-fold diluted blood at a ratio of 1 LN229 cell to 10,000 RBCs. To account for the small population of LN229 cells, we analyzed 200,000 cells to identify a significant cluster of this cell type.

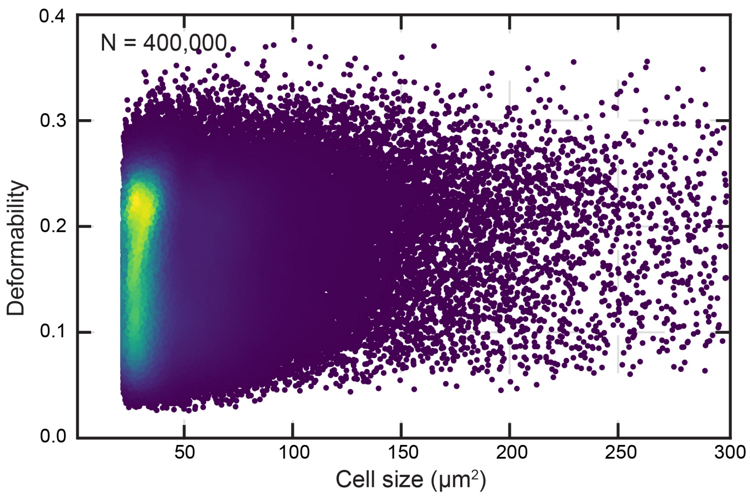

Fig. S16. Whole blood analysis in real time. Analysis of 20-fold diluted whole blood, with cells being deformed as they pass through a 15 µm × 15 µm cross section microfluidic channel at velocities exceeding 0.7 m/s.

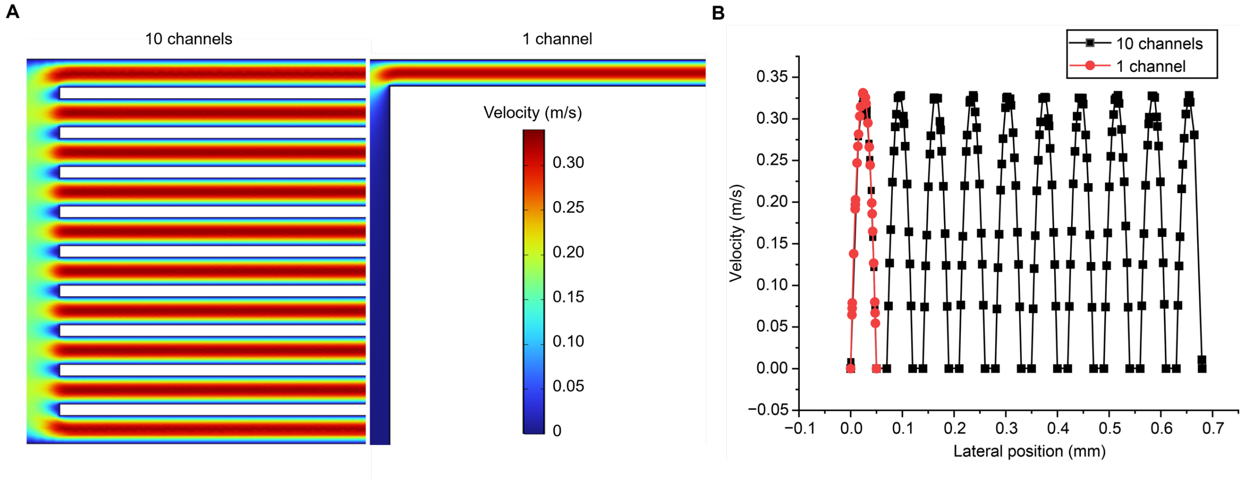

Fig. S17. Numerical simulation comparing flow field distribution using 10 channels versus one channel. A) Computational model depicting the flow field distribution in a microfluidic device with 10 parallel channels versus a single-channel configuration. B) Velocity profile comparison between the 10-channel and single-channel configurations shows consistent flow characteristics despite variations in channel blockage. The use of a pressure pump as a source maintains uniform velocity fields across channels, ensuring consistent fluid dynamics even under conditions of channel obstruction.

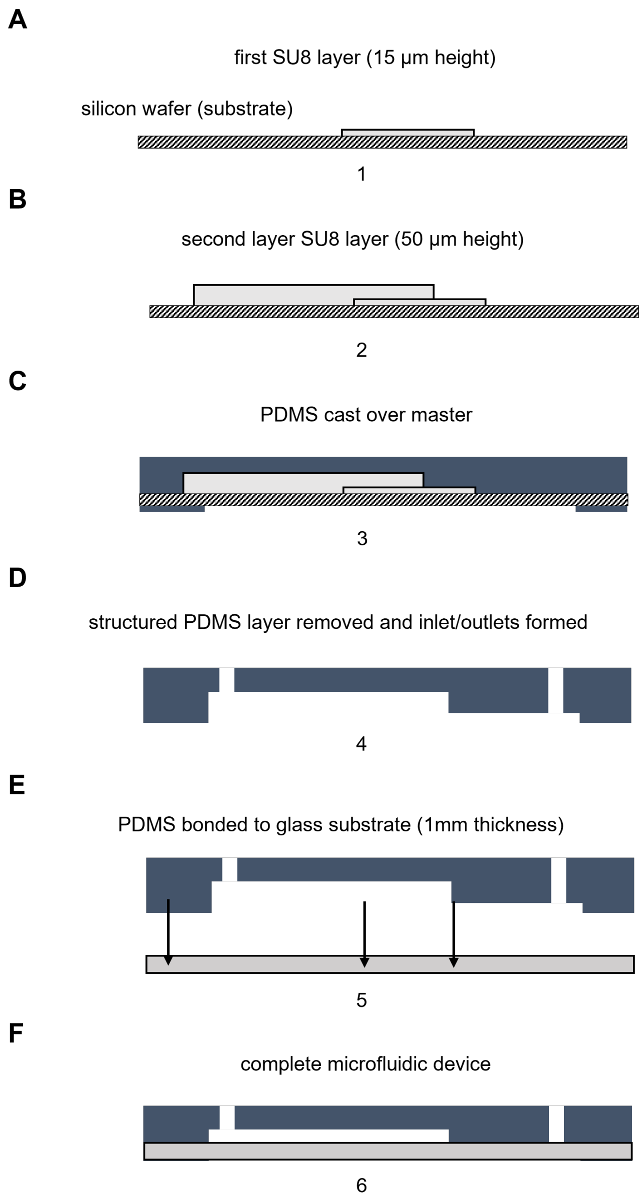

Fig. S18. Schematic of the workflow for the fabrication of the two-layer PDMS microfluidic device. A) The first SU8 layer (15 µm high) is deposited on a silicon wafer. B) The second SU8 layer, having a 50-µm height is formed next to the first layer. C) PDMS is cast over the multilayer master mold and subsequently cured. D) The structured PDMS layer is peeled from the master mold and inlet/outlet ports created. E) The structured PDMS layer is plasma-bonded to a flat glass slide. F) A cross-sectional view of the completed microfluidic device.

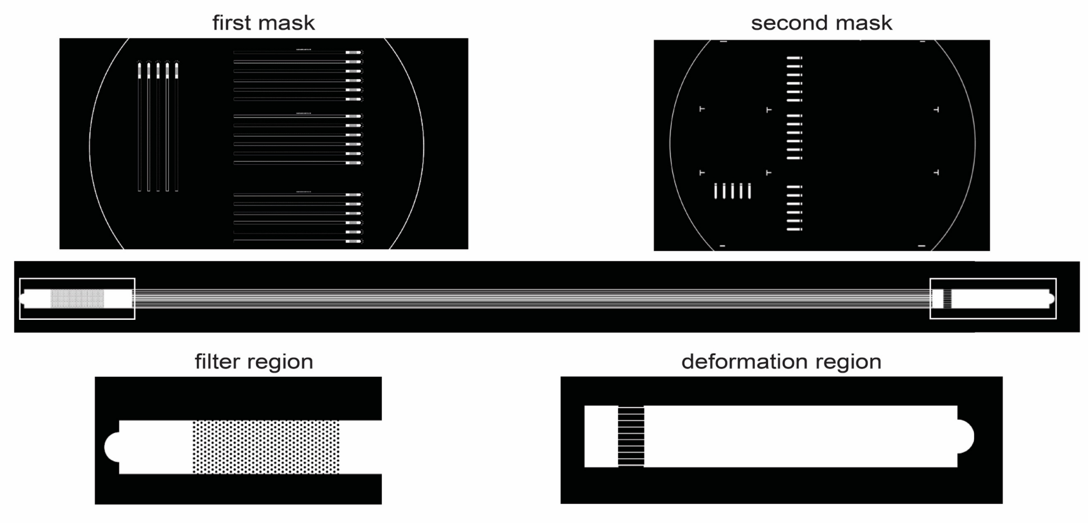

Fig. S19. Two mask patterns designed using AutoCAD 2019. The first mask defines the dimensions of the focusing and relaxation zones, whilst the second mask defines the structure of the deformation zone.

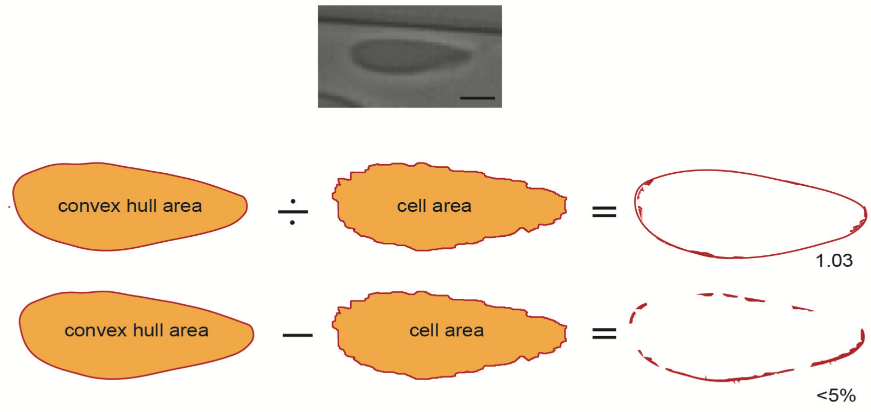

Fig. S20. Graphical representation of the cell area ratio and difference. Cells with an area ratio below 1.05 or an area difference less than 5%, have a convex contour. Cells with area ratio above 1.05 or an area difference more than 5% are filtered out. Representative image of a cell with an area ratio of 1.03. Scale bar:10 μm.

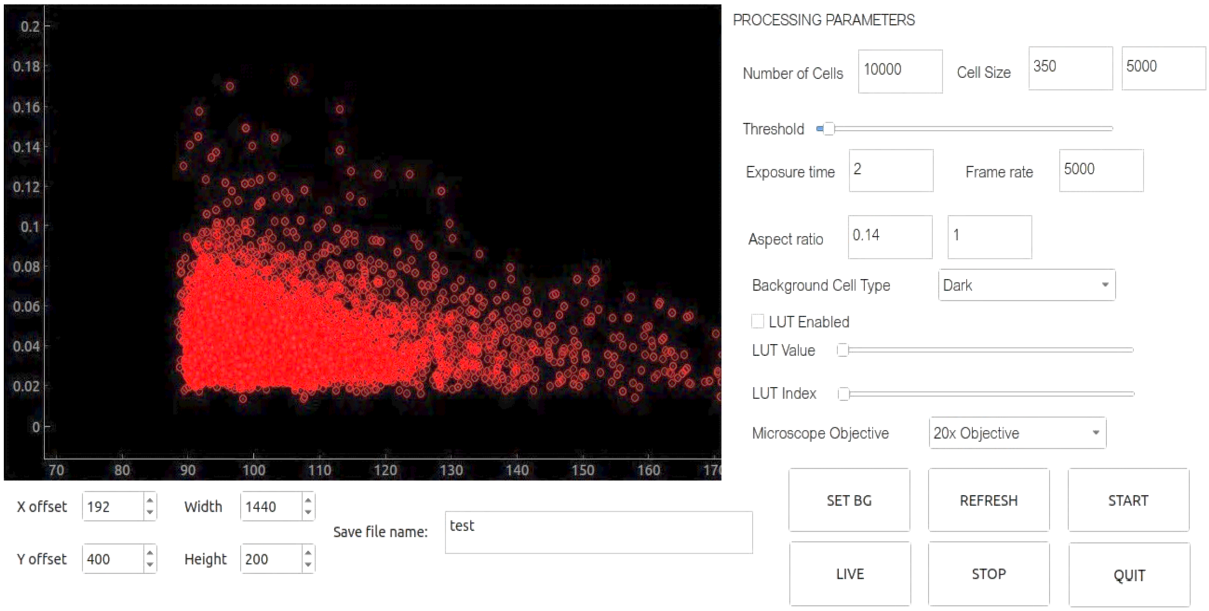

Fig. S21. GUI of the video processing software for real-time deformability cytometry. Input parameters such as the number of cells to be analyzed, cell size, the threshold as a criterion of image binarization, exposure time, camera frame rate, the cell aspect ratio and image background type are user defined. Data are displayed in a 2D scatterplot, showing deformability against cross-sectional area. For each cell additional parameters such as the cell size and mean brightness are exported for further analysis.

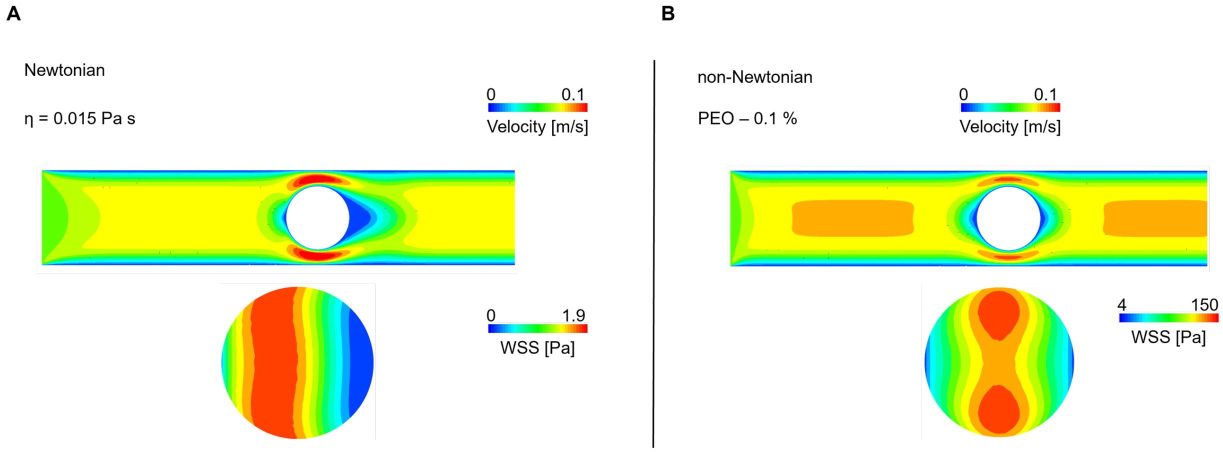

Fig. S22. Analysis of the impact of fluid properties on the wall shear stress (WSS) under no slip boundary conditions. A cell with a diameter of 10 µm is positioned at the center of a 15 µm channel. Simulations were conducted using a Newtonian fluid (A) and 0.1% 1MDa PEO viscoelastic fluid (B) to determine the distribution of wall shear stress around the cell. A fixed flow rate of 3 µl/s was used for both scenarios. WSS values and their associated distribution patterns are different for the Newtonian and viscoelastic fluid.

Table S1: Viscoelastic properties of different PEO concentration fluids. These values were obtained by curve fitting of the viscosity plots at different concentrations of PEO 1MDa (Fig. S8b) using the Carreau model.

PEO 1MDa	μ_0 (mPa.s)	μ_{inf} (mPa.s)	λ (s)	n
0.1% (w/v)	1.55	1.40	21.22	0.8107
0.5% (w/v)	4.05	3.98	18.37	0.8541
0.8% (w/v)	7.80	7.40	0.007	0.1044
1.0% (w/v)	26	18	0.009	0.8204

Table S2. Conventional flow cytometry and vDC data for rare cell detection experiments. LN229 cells were spiked in blood at ratios of 1:1,000 and 1:10,000 (LN229: RBCs)

**prepared ratios**	**conventional flow cytometry**			**vDC**
LN229 : Blood Cells	LN229 per μL	Blood cells per μL	Ratio LN229 : Blood Cells	Ratio LN229 : Blood Cells
1:1,000	5.520	4037	0.0014	0.0016
1:10,000	0.610	4128	0.00018	0.00022

Table S3. Solver settings of the computational simulations. These simulation setting were used to evaluate the distribution of hemodynamic shear stress on the cell surface due to fluid viscoelasticity.

Type	Pressure-based Ansys Fluent
Pressure velocity coupling method	coupled
Spatial discretization scheme	
gradient	least squares cell based
pressure	second order
momentum	second order upwind
Steady state formulation	second order implicit
**Relaxation factors**	
Flow Courant number	50
Explicit relaxation factors	
momentum	0.35
pressure	0.35
**Convergence criterion residuals**	
continuity	5×10^{-5}
velocity	5×10^{-5}
max iterations	1000

Movie S1. High-speed imaging of the cell focusing and deformability process of the VDC device.

REFERENCES AND NOTES

1. E. Kilgour, D. G. Rothwell, G. Brady, C. Dive, Liquid biopsy-based biomarkers of treatment response and resistance. *Cancer Cell* **37**, 485–495 (2020).

2. S. M. Batool, A. Yekula, P. Khanna, T. Hsia, A. S. Gamblin, E. Ekanayake, A. K. Escobedo, D. G. You, C. M. Castro, H. Im, T. Kilic, M. A. Garlin, J. Skog, D. M. Dinulescu, J. Dudley, N. Agrawal, J. Cheng, F. Abtin, D. R. Aberle, D. Chia, D. Elashoff, T. Grognan, K. Krysan, S. S. Oh, C. Strom, M. Tu, F. Wei, R. R. Xian, S. J. Skates, D. Y. Zhang, T. Trinh, M. Watson, R. Aft, S. Rawal, A. Agarwal, S. B. Kesmodel, C. Yang, C. Shen, F. H. Hochberg, D. T. W. Wong, A. A. Patel, N. Papadopoulos, C. Bettegowda, R. J. Cote, S. Srivastava, H. Lee, B. S. Carter, L. Balaj, The Liquid Biopsy Consortium: Challenges and opportunities for early cancer detection and monitoring. *Cell Rep. Med.* **4**, 101198 (2023).

3. C. T. Mierke, Viscoelasticity, like forces, plays a role in mechanotransduction. *Front. Cell Dev. Biol.* **10**, 789841 (2022).

4. T. Bongiorno, J. L. Chojnowski, J. D. Lauderdale, T. Sulchek, Cellular stiffness as a novel stemness marker in the corneal limbus. *Biophys. J.* **111**, 1761–1772 (2016).

5. H. T. Tse, D. R. Gossett, Y. S. Moon, M. Masaeli, M. Sohsman, Y. Ying, K. Mislick, R. P. Adams, J. Rao, D. Di Carlo, Quantitative diagnosis of malignant pleural effusions by single-cell mechanophenotyping. *Sci. Transl. Med.* **5**, 212ra163 (2013).

6. J. Guck, S. Schinkinger, B. Lincoln, F. Wottawah, S. Ebert, M. Romeyke, D. Lenz, H. M. Erickson, R. Ananthakrishnan, D. Mitchell, J. Käs, S. Ulvick, C. Bilby, Optical deformability as an inherent cell marker for testing malignant transformation and metastatic competence. *Biophys. J.* **88**, 3689–3698 (2005).

7. S. C. Hur, N. K. Henderson-MacLennan, E. R. B. McCabe, D. Di Carlo, Deformability-based cell classification and enrichment using inertial microfluidics. *Lab Chip* **11**, 912–920 (2011).

8. V. Swaminathan, K. Mythreye, E. T. O'Brien, A. Berchuck, G. C. Blobe, R. Superfine, Mechanical stiffness grades metastatic potential in patient tumor cells and in cancer cell lines. *Cancer Res.* **71**, 5075–5080 (2011).

9. H. W. Hou, Q. S. Li, G. Y. Lee, A. P. Kumar, C. N. Ong, C. T. Lim, Deformability study of breast cancer cells using microfluidics. *Biomed. Microdevices* **11**, 557–564 (2009).

10. Physical Sciences - Oncology Centers Network, D. B. Agus, J. F. Alexander, W. Arap, S. Ashili, J. E. Aslan, R. H. Austin, V. Backman, K. J. Bethel, R. Bonneau, W.-C. Chen, C. Chen-Tanyolac, N. C. Choi, S. A. Curley, M. Dallas, D. Damania, P. C. W. Davies, P. Decuzzi, L. Dickinson, L. Estevez-Salmeron, V. Estrella, M. Ferrari, C. Fischbach, J. Foo, S. I. Fraley, C. Frantz, A. Fuhrmann, P. Gascard, R. A. Gatenby, Y. Geng, S. Gerecht, R. J. Gillies, B. Godin, W. M. Grady,

A. Greenfield, C. Hemphill, B. L. Hempstead, A. Hielscher, W D. Hillis, E. C. Holland, A. Ibrahim-Hashim, T. Jacks, R. H. Johnson, A. Joo, J. E. Katz, L. Kelbauskas, C. Kesselman, M. R. King, K. Konstantopoulos, C. M. Kraning-Rush, P. Kuhn, K. Kung, B. Kwee, J. N. Lakins, G. Lambert, D. Liao, J. D. Licht, J. T. Liphardt, L. Liu, M. C. Lloyd, A. Lyubimova, P. Mallick, J. Marko, O. J. T. Mc Carty, D. R. Meldrum, F. Michor, S. M. Mumenthaler, V. Nandakumar, T. V. O'Halloran, S. Oh, R. Pasqualini, M. J. Paszek, K. G. Philips, C. S. Poultney, K. Rana, C. A. Reinhart-King, R. Ros, G. L. Semenza, P. Senechal, M. L. Shuler, S. Srinivasan, J. R. Staunton, Y. Stypula, H. Subramanian, T. D. Tlsty, G. W. Tormoen, Y. Tseng, A. van Oudenaarden, S. S. Verbridge, J. C. Wan, V. M. Weaver, J. Widom, C. Will, D. Wirtz, J. Wojtkowiak, P.-H. Wu, A physical sciences network characterization of non-tumorigenic and metastatic cells. *Sci. Rep.* **3**, 1449 (2013).

11. M. Radmacher, Studying the mechanics of cellular processes by atomic force microscopy. *Method. Cell Biol.* **83**, 347–372 (2007).

12. P. Preira, V. Grandné, J. M. Forel, S. Gabriele, M. Camara, O. Theodoly, Passive circulating cell sorting by deformability using a microfluidic gradual filter. *Lab Chip* **13**, 161–170 (2013).

13. R. M. Hochmuth, Micropipette aspiration of living cells. *J. Biomech.* **33**, 15–22 (2000).

14. J. Guck, R. Ananthakrishnan, H. Mahmood, T. J. Moon, C. C. Cunningham, J. Käs, The optical stretcher: A novel laser tool to micromanipulate cells. *Biophys. J.* **81**, 767–784 (2001).

15. G. Jiang, G. Giannone, D. R. Critchley, E. Fukumoto, M. P. Sheetz, Two-piconewton slip bond between fibronectin and the cytoskeleton depends on talin. *Nature* **424**, 334–337 (2003).

16. E. M. Darling, D. D. Carlo, High-throughput assessment of cellular mechanical properties. *Annu. Rev. Biomed. Eng.* **17**, 35–62 (2015).

17. K. D. Nyberg, K. H. Hu, S. H. Kleinman, D. B. Khismatullin, M. J. Butte, A. C. Rowat, Quantitative deformability cytometry: Rapid, calibrated measurements of cell mechanical properties. *Biophys. J.* **113**, 1574–1584 (2017).

18. A. Adamo, A. Sharei, L. Adamo, B. Lee, S. Mao, K. F. Jensen, Microfluidics-based assessment of cell deformability. *Anal. Chem.* **84**, 6438–6443 (2012).

19. M. J. Rosenbluth, W. A. Lam, D. A. Fletcher, Analyzing cell mechanics in hematologic diseases with microfluidic biophysical flow cytometry. *Lab Chip* **8**, 1062–1070 (2008).

20. S. Byun, S. Son, D. Amodei, N. Cermak, J. Shaw, J. H. Kang, V. C. Hecht, M. M. Winslow, T. Jacks, P. Mallick, S. R. Manalis, Characterizing deformability and surface friction of cancer cells. *Proc. Natl. Acad. Sci. U.S.A.* **110**, 7580–7585 (2013).

21. O. Otto, P. Rosendahl, A. Mietke, S. Golfier, C. Herold, D. Klaue, S. Girardo, S. Pagliara, A. Ekpenyong, A. Jacobi, M. Wobus, N. Töpfner, U. F. Keyser, J. Mansfeld, E. Fischer-Friedrich, J.

Guck, Real-time deformability cytometry: On-the-fly cell mechanical phenotyping. *Nat. Methods* **12**, 199–202 (2015).

22. A. A. Nawaz, M. Urbanska, M. Herbig, M. Nötzel, M. Kräter, P. Rosendahl, C. Herold, N. Toepfner, M. Kubánková, R. Goswami, S. Abuhattum, F. Reichel, P. Müller, A. Taubenberger, S. Girardo, A. Jacobi, J. Guck, Intelligent image-based deformation-assisted cell sorting with molecular specificity. *Nat. Methods* **17**, 595–599 (2020).

23. P. Rosendahl, K. Plak, A. Jacobi, M. Kraeter, N. Toepfner, O. Otto, C. Herold, M. Winzi, M. Herbig, Y. Ge, S. Girardo, K. Wagner, B. Baum, J. Guck, Real-time fluorescence and deformability cytometry. *Nat. Methods* **15**, 355–358 (2018).

24. M. Piergiovanni, V. Galli, G. Holzner, S. Stavrakis, A. DeMello, G. Dubini, Deformation of leukaemia cell lines in hyperbolic microchannels: Investigating the role of shear and extensional components. *Lab Chip* **20**, 2539–2548 (2020).

25. M. Mokbel, D. Mokbel, A. Mietke, N. Träber, S. Girardo, O. Otto, J. Guck, S. Aland, Numerical simulation of real-time deformability cytometry to extract cell mechanical properties. *ACS Biomater Sci. Eng.* **3**, 2962–2973 (2017).

26. D. R. Gossett, H. T. K. Tse, S. A. Lee, Y. Ying, A. G. Lindgren, O. O. Yang, J. Rao, A. T. Clark, D. Di Carlo, Hydrodynamic stretching of single cells for large population mechanical phenotyping. *Proc. Natl. Acad. Sci. U.S.A.* **109**, 7630–7635 (2012).

27. L. Guillou, J. B. Dahl, J. G. Lin, A. I. Barakat, J. Husson, S. J. Muller, S. Kumar, Measuring cell viscoelastic properties using a microfluidic extensional flow device. *Biophys. J.* **111**, 2039–2050 (2016).

28. M. Asghari, X. Cao, B. Mateescu, D. van Leeuwen, M. K. Aslan, S. Stavrakis, A. J. deMello, Oscillatory viscoelastic microfluidics for efficient focusing and separation of nanoscale species. *ACS Nano* **14**, 422–433 (2020).

29. G. Holzner, S. Stavrakis, A. deMello, Elasto-inertial focusing of mammalian cells and bacteria using low molecular, low viscosity PEO solutions. *Anal. Chem.* **89**, 11653–11663 (2017).

30. X. Lu, C. Liu, G. Hu, X. Xuan, Particle manipulations in non-Newtonian microfluidics: A review. *J. Colloid Interface Sci.* **500**, 182–201 (2017).

31. S. Cha, T. Shin, S. S. Lee, W. Shim, G. Lee, S. J. Lee, Y. Kim, J. M. Kim, Cell stretching measurement utilizing viscoelastic particle focusing. *Anal. Chem.* **84**, 10471–10477 (2012).

32. Y. B. Bae, H. K. Jang, T. H. Shin, G. Phukan, T. T. Tran, G. Lee, W. R. Hwang, J. M. Kim, Microfluidic assessment of mechanical cell damage by extensional stress. *Lab Chip* **16**, 96–103 (2016).

33. D. Yuan, Q. Zhao, S. Yan, S.-Y. Tang, G. Alici, J. Zhang, W. Li, Recent progress of particle migration in viscoelastic fluids. *Lab Chip* **18**, 551–567 (2018).

34. L. E. Rodd, J. J. Cooper-White, D. V. Boger, G. H. McKinley, Role of the elasticity number in the entry flow of dilute polymer solutions in micro-fabricated contraction geometries. *J. Nonnewton. Fluid. Mech.* **143**, 170–191 (2007).

35. G. Holzner, B. Mateescu, D. van Leeuwen, G. Cereghetti, R. Dechant, S. Stavrakis, A. deMello, High-throughput multiparametric imaging flow cytometry: Toward diffraction-limited sub-cellular detection and monitoring of sub-cellular processes. *Cell Rep.* **34**, 108824 (2021).

36. Y. Chen, L. Jiang, X. Zhang, Z. Ni, N. Xiang, Viscoelastic-sorting integrated deformability cytometer for high-throughput sorting and high-precision mechanical phenotyping of tumor cells. *Anal. Chem.* **95**, 18180–18187 (2023).

37. A. De Mello, X. Cao, M. Asghari, M. Aslan, B. Mateescu, Y. Meng, Method and microfluidic device for studying cell deformations. Patent WO2022229011A1 (2022).

38. D. Soteriou, M. Kubánková, C. Schweitzer, R. López-Posadas, R. Pradhan, O.-M. Thoma, A.-H. Györfi, A.-E. Matei, M. Waldner, J. H. W. Distler, S. Scheuermann, J. Langejürgen, M. Eckstein, R. Schneider-Stock, R. Atreya, M. F. Neurath, A. Hartmann, J. Guck, Rapid single-cell physical phenotyping of mechanically dissociated tissue biopsies. *Nat. Biomed. Eng.* **7**, 1392–1403 (2023).

39. H. R. O'Neal Jr, R. Sheybani, D. R. Janz, R. Scoggins, T. Jagneaux, J. E. Walker, D. J. Henning, E. Rosenman, S. A. Mahler, H. Regunath, C. S. Sampson, D. C. Files, R. D. Fremont, M. J. Noto, E. E. Schneider, W. R. Shealey, M. S. Berlinger, T. C. Carver, M. K. Walker, N. A. Ledeboer, A. M. Shah, H. T. K. Tse, D. DiCarlo, T. W. Rice, C. B. Thomas, Validation of a novel, rapid sepsis diagnostic for emergency department use. *Crit. Care Explor.* **6**, e1026 (2024).

40. H. R. O'Neal Jr, R. Sheybani, T. S. Caffery, M. W. Musso, D. Hamer. S. M. Alwood, M.S. Berlinger, T. Jagneaux, K. W. LaVie, C. S. O'Neal, M. A. Sanchez, M. K Walker, A. M. Shah, H. T. K. Tse, C. B. Thomas, Assessment of a cellular host response test as a sepsis diagnostic for those with suspected infection in the emergency department. *Crit. Care Explor.* **3** e0460 (2021).

41. M. Urbanska, H. E. Muñoz, J. Shaw Bagnall, O. Otto, S. R. Manalis, D. Di Carlo, J. Guck, A comparison of microfluidic methods for high-throughput cell deformability measurements. *Nat. Methods* **17**, 587–593 (2020).

42. S. Golfier, P. Rosendahl, A. Mietke, M. Herbig, J. Guck, O. Otto, High-throughput cell mechanical phenotyping for label-free titration assays of cytoskeletal modifications. *Cytoskeleton* **74**, 283–296 (2017).

43. J. Swift, I. L. Ivanovska, A. Buxboim, T. Harada, P. C. Dingal, J. Pinter, J. D. Pajerowski, K. R. Spinler, J. W. Shin, M. Tewari, F. Rehfeldt, D. W. Speicher, D. E. Discher, Nuclear lamin-A scales with tissue stiffness and enhances matrix-directed differentiation. *Science* **341**, 1240104 (2013).

44. Z. Tariq, H. Zhang, A. Chia-Liu, Y. Shen, Y. Gete, Z. M. Xiong, C. Tocheny, L. Campanello, D. Wu, W. Losert, K. Cao, Lamin A and microtubules collaborate to maintain nuclear morphology. *Nucleus* **8**, 433–446 (2017).

45. H. Kubitschke, J. Schnauss, K. D. Nnetu, E. Warmt, R. Stange, J. Kaes, Actin and microtubule networks contribute differently to cell response for small and large strains. *New J. Phys.* **19**, 093003 (2017).

46. T. Harada, J. Swift, J. Irianto, J. W. Shin, K. R. Spinler, A. Athirasala, R. Diegmiller, P. C. Dingal, I. L. Ivanovska, D. E. Discher, Nuclear lamin stiffness is a barrier to 3D migration, but softness can limit survival. *J. Cell Biol.* **204**, 669–682 (2014).

47. C. Müller, J. Holtschmidt, M. Auer, E. Heitzer, K. Lamszus, A. Schulte, J. Matschke, S. Langer-Freitag, C. Gasch, M. Stoupiec, O. Mauermann, S. Peine, M. Glatzel, M. R. Speicher, J. B. Geigl, M. Westphal, K. Pantel, S. Riethdorf, Hematogenous dissemination of glioblastoma multiforme. *Sci. Transl. Med.* **6**, 247ra101 (2014).

48. Q. T. Ostrom, M. Price, C. Neff, G. Cioffi, K. A. Waite, C. Kruchko, J. S. Barnholtz-Sloan, CBTRUS statistical report: Primary brain and other central nervous system tumors diagnosed in the United States in 2015-2019. *Neuro Oncol.* **24**, v1–v95 (2022).

49. E. Jung, M. Osswald, M. Ratliff, H. Dogan, R. Xie, S. Weil, D. C. Hoffmann, F. T. Kurz, T. Kessler, S. Heiland, A. von Deimling, F. Sahm, W. Wick, F. Winkler, Tumor cell plasticity, heterogeneity, and resistance in crucial microenvironmental niches in glioma. *Nat. Commun.* **12**, 1014 (2021).

50. J. D. Lathia, S. C. Mack, E. E. Mulkearns-Hubert, C. L. Valentim, J. N. Rich, Cancer stem cells in glioblastoma. *Genes Dev.* **29**, 1203–1217 (2015).

51. J. D. Lathia, J. Gallagher, J. T. Myers, M. Li, A. Vasanji, R. E. McLendon, A. B. Hjelmeland, A. Y. Huang, J. N. Rich, Direct in vivo evidence for tumor propagation by glioblastoma cancer stem cells. *PLOS ONE* **6**, e24807 (2011).

52. S. Bao, Q. Wu, R. E. McLendon, Y. Hao, Q. Shi, A. B. Hjelmeland, M. W. Dewhirst, D. D. Bigner, J. N. Rich, Glioma stem cells promote radioresistance by preferential activation of the DNA damage response. *Nature* **444**, 756–760 (2006).

53. X. Gong, P. H. Schwartz, M. E. Linskey, D. A. Bota, Neural stem/progenitors and glioma stem-like cells have differential sensitivity to chemotherapy. *Neurology* **76**, 1126–1134 (2011).

54. A. Dirkse, A. Golebiewska, T. Buder, P. V. Nazarov, A. Muller, S. Poovathingal, N. H. C. Brons, S. Leite, N. Sauvageot, D. Sarkisjan, M. Seyfrid, S. Fritah, D. Stieber, A. Michelucci, F. Hertel, C.

Herold-Mende, F. Azuaje, A. Skupin, R. Bjerkvig, A. Deutsch, A. Voss-Böhme, S. P. Niclou, Stem cell-associated heterogeneity in glioblastoma results from intrinsic tumor plasticity shaped by the microenvironment. *Nat. Commun.* **10**, 1787 (2019).

55. K. Lenting, R. Verhaak, M. ter Laan, P. Wesseling, W. Leenders, Glioma: Experimental models and reality. *Acta Neuropathol.* **133**, 263–282 (2017).

56. J. A. Innes, A. S. Lowe, R. Fonseca, N. Aley, T. El-Hassan, M. Constantinou, J. Lau, A. Eddaoudi, S. Marino, S. Brandner, Phenotyping clonal populations of glioma stem cell reveals a high degree of plasticity in response to changes of microenvironment. *Lab. Invest.* **102**, 172–184 (2022).

57. W. Yu, S. Sharma, E. Rao, A. C. Rowat, J. K. Gimzewski, D. Han, J. Rao, Cancer cell mechanobiology: A new frontier for cancer research. *J. Natl. Cancer Inst.* **2**, 10–17 (2022).

58. J. V. Melo, D. Catovsky, W. M. Gregory, D. A. Galton, The relationship between chronic lymphocytic leukaemia and prolymphocytic leukaemia. IV. Analysis of survival and prognostic features. *Br. J. Haematol.* **65**, 23–29 (1987).

59. M. Stetler-Stevenson, Flow cytometry in lymphoma diagnosis and prognosis: Useful? *Best Pract. Res. Clin. Haematol.* **16**, 583–597 (2003).

60. M. A. Chowdury, K. L. Heileman, T. A. Moore, E. W. K. Young, Biomicrofluidic systems for hematologic cancer research and clinical applications. *SLAS Technol.* **24**, 457–476 (2019).

61. M. J. Stuart, R. L. Nagel, Sickle-cell disease. *The Lancet* **364**, 1343–1360 (2004).

62. J. A. Chasis, S. L. Schrier, Membrane deformability and the capacity for shape change in the erythrocyte. *Blood* **74**, 2562–2568 (1989).

63. S. L. Schrier, Thalassemia: Pathophysiology of red cell changes. *Annu. Rev. Med.* **45**, 211–218 (1994).

64. E. Llaudet-Planas, J. L. Vives-Corrons, V. Rizzuto, P. Gomez-Ramirez, J. S. Navarro, M. T. C. Sibina, M. Garcia-Bernal, A. R. Llobet, I. Badell, P. Velasco-Puyo, J. L. Dapena, M. M. Manu-Pereira, Osmotic gradient ektacytometry: A valuable screening test for hereditary spherocytosis and other red blood cell membrane disorders. *Int. J. Lab. Hematol.* **40**, 94–102 (2018).

65. L. Admoni-Elisha, I. Nakdimon, A. Shteinfer, T. Prezma, T. Arif, N. Arbel, A. Melkov, O. Zelichov, I. Levi, V. Shoshan-Barmatz, Novel biomarker proteins in chronic lymphocytic leukemia: Impact on diagnosis, prognosis and treatment. *PLOS ONE* **11**, e0148500 (2016).

66. D. Di Carlo, A mechanical biomarker of cell state in medicine. *J. Lab Autom.* **17**, 32–42 (2012).

67. M. K. Aslan, Y. Meng, Y. Zhang, T. Weiss, S. Stavrakis, A. J. deMello, Ultrahigh-throughput, real-time flow cytometry for rare cell quantification from whole blood. *ACS Sens.* **9**, 474–482 (2024).

68. T. Weiss, H. Schneider, M. Silginer, A. Steinle, M. Pruschy, B. Polić, M. Weller, P. Roth, NKG2D-dependent antitumor effects of chemotherapy and radiotherapy against glioblastoma. *Clin. Cancer Res.* **24**, 882–895 (2018).

69. K. Hood, S. Lee, M. Roper, Inertial migration of a rigid sphere in three-dimensional Poiseuille flow. *J. Fluid Mech.* **765**, 452–479 (2015).

70. K. Hood, S. Kahkeshani, D. Di Carlo, M. Roper, Direct measurement of particle inertial migration in rectangular microchannels. *Lab Chip* **16**, 2840–2850 (2016).

71. K. Kang, S. S. Lee, K. Hyun, S. J. Lee, J. M. Kim, DNA-based highly tunable particle focuser. *Nat. Commun.* **4**, 2567 (2013).

72. D. Li, X. Lu, X. Xuan, Viscoelastic separation of particles by size in straight rectangular microchannels: A parametric study for a refined understanding. *Anal. Chem.* **88**, 12303–12309 (2016).

73. Y. Bazilevs, T. J. R. Hughes, Weak imposition of Dirichlet boundary conditions in fluid mechanics. *Comput. Fluids* **36**, 12–26 (2007).

74. K. S. Rao, K. G. Sravani, G. Yugandhar, G. V. Rao, V. N. Mani, Design and analysis of fluid structure interaction in a horizontal micro channel. *Procedia Mater. Sci.* **10**, 768–788 (2015).

75. L. D. Wittwer, F. Reichel, P. Müller, J. Guck, S. Aland, A new hyperelastic lookup table for RT-DC. *J. Soft Matter* **19**, 2064–2073 (2023).
